# Tailoring Diffusional Fields in Zwitterion/Dopamine Copolymer Electropolymerized at Carbon Nanowalls for Sensitive Recognition of Neurotransmitters

**DOI:** 10.1021/acsnano.2c06406

**Published:** 2022-07-22

**Authors:** Adrian Olejnik, Mateusz Ficek, Marek Szkodo, Alicja Stanisławska, Jakub Karczewski, Jacek Ryl, Anna Dołęga, Katarzyna Siuzdak, Robert Bogdanowicz

**Affiliations:** † Department of Metrology and Optoelectronics, Faculty of Electronics, Telecommunications and Informatics, Gdańsk University of Technology, Narutowicza 11/12 St., 80-233 Gdańsk, Poland; ‡ Centre for Plasma and Laser Engineering, The Szewalski Institute of Fluid-Flow Machinery, Polish Academy of Sciences, Fiszera 14 St., 80-231 Gdańsk, Poland; § Institute of Manufacturing and Materials Technology, Faculty of Mechanical Engineering and Ship Technology, Gdańsk University of Technology, Narutowicza 11/12 St., 80-233 Gdańsk, Poland; ∥ Institute of Nanotechnology and Materials Engineering and Advanced Materials Center, Gdańsk University of Technology, Narutowicza 11/12, 80-233 Gdansk, Poland; ⊥ Department of Inorganic Chemistry, Faculty of Chemistry, Gdańsk University of Technology, Narutowicza 11/12 St., 80-233 Gdańsk, Poland

**Keywords:** polydopamine, zwitterions, coelectropolymerization, carbon nanowalls, nanoindentation, sensing, diffusion fields

## Abstract

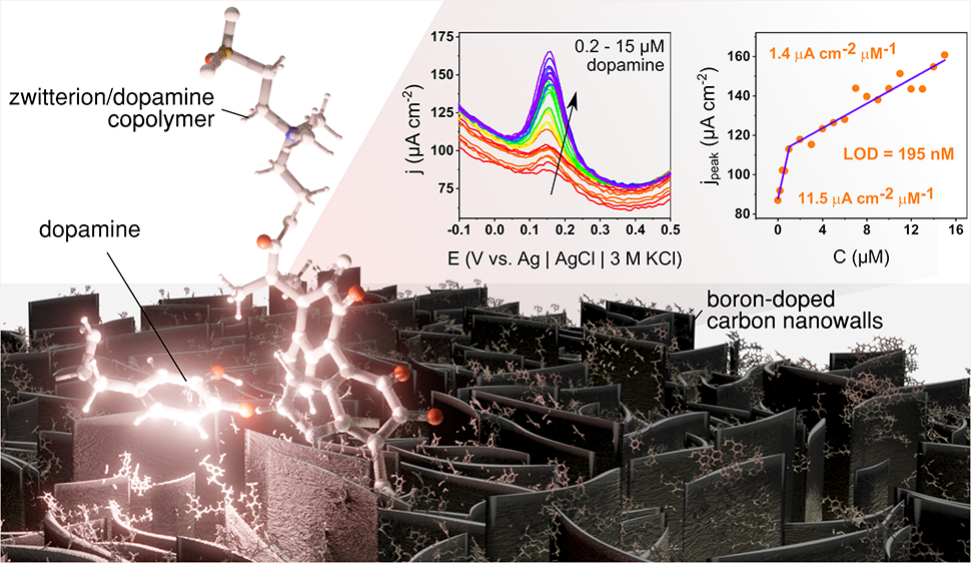

The importance of neurotransmitter sensing in the diagnosis and treatment of many psychological illnesses and neurodegenerative diseases is non-negotiable. For electrochemical sensors to become widespread and accurate, a long journey must be undertaken for each device, from understanding the materials at the molecular level to real applications in biological fluids. We report a modification of diamondized boron-doped carbon nanowalls (BCNWs) with an electropolymerized polydopamine/polyzwitterion (PDA|PZ) coating revealing tunable mechanical and electrochemical properties. Zwitterions are codeposited with PDA and noncovalently incorporated into a structure. This approach causes a specific separation of the diffusion fields generated by each nanowall during electrochemical reactions, thus increasing the contribution of the steady-state currents in the amperometric response. This phenomenon has a profound effect on the sensing properties, leading to a 4-fold enhancement of the sensitivity (3.1 to 14.3 μA cm^–2^ μM^–1^) and a 5-fold decrease of the limit of detection (505 to 89 nM) in comparison to the pristine BCNWs. Moreover, as a result of the antifouling capabilities of the incorporated zwitterions, this enhancement is preserved in bovine serum albumin (BSA) with a high protein concentration. The presence of zwitterion facilitates the transport of dopamine in the direction of the electrode by intermolecular interactions such as cation−π and hydrogen bonds. On the other hand, polydopamine units attached to the surface form molecular pockets driven by hydrogen bonds and π–π interactions. As a result, the intermediate state of dopamine–analyte oxidation is stabilized, leading to the enhancement of the sensing properties.

## Introduction

The number of patients with mental illnesses and neurodegenerative diseases is rapidly increasing all over the world. Many of these diseases are associated with dysfunctions in dopaminergic transmission centers in the brain. In particular, a deficiency in the substantia nigra is typical in Parkinson’s disease and leads to motor dysfunctions such as tremors.^1^ Attention deficit disorder (ADD) is associated with dysregulation of noradrenaline metabolism in the locus coeruleus and dopaminergic transmission in the mesolimbic and mesocortical pathways, which cause impairment of executive functions, memory, and concentration.^2,3^ On the other hand, the long-standing hypothesis of schizophrenia involves excessive firing of dopaminergic neurons in the stratum, leading to positive symptoms such as paranoia.^4^

Engineering directed medical therapies as well as understanding the neurological and metabolic background of those diseases requires precise and inexpensive tools for in situ measurements of dopamine concentrations in the synaptic clefts and interstitial fluid of neural tissue.^5^ The most potent advantage of the electrochemical sensors of neurotransmitters and neuromodulators is their very high temporal (time) resolution compared to fluorescent sensors.^5^ Moreover, this method of sensing does not require the introduction of carefully engineered viral vectors (e.g., adenoviruses) capable of expressing the desired fluorescent markers into the tissue.

However, the most critical drawback of electrochemical sensors is their relatively low temporal (spatial) resolution,^5^ which unfortunately does not allow precise imaging of single-neuron transmission and modulation. A potential solution to this problem is the fabrication of sensitive microelectrodes that are able to accomplish this task.^6^ Therefore, it is essential to fabricate miniaturizable electrode materials with a very high affinity toward sensing a specific molecule, i.e., high sensitivity and selectivity, low limit of detection, and the ability to resist biofouling.

Polyzwitterions (PZs) are polymers possessing two ionized functional groups in each structural unit: one positive and one negative.^7^ They are well-known due to their antifouling properties,^8−12^ i.e., their ability to resist the adsorption of various biomolecules and to prevent the creation of biofilm on their surface. The antifouling mechanisms are generally complicated. Briefly, due to dipole–dipole and dipole–multipole interactions, the zwitterion-coated surface has a high level of hydration with a low ion concentration in the double layer. In this case, the adsorption of a protein is thermodynamically unfavorable, because there is neither entropic gain through counterion release nor enthalpic gain through reduction of the contact surface area.^10,12^ Therefore, polyzwitterions are potent candidates for coatings for biosensors.

Dopamine can be polymerized on various surfaces using oxidative polymerization or electropolymerization.^13−17^ The structure of the resulting polydopamine (PDA) consists of several chemically different structural units arranged in covalently or noncovalently cross-linked chains.^18,19^ Among these, there are single-ringed dopamine (DA) and dopamine quinone (DQ) units containing the primary amine group as well as double-ringed dihydroxyindole (DHI) and indole quinone (IQ) units with aromatic carbon atoms capable of forming aryl–aryl cross-links.^19,20^ PDA has a set of very useful properties due to its capability of forming strong hydrogen bonds and π–π and π–cation interactions with various molecules.^18,21^ It makes PDA a superior adhesive promoting agent,^22^ complexation agent for chelating metal ions,^23,24^ template for molecular imprinting,^25^ and a functional ingredient of stimuli (pH, temperature, electric field, mechanical) responsive coatings^26^ and supercapacitors.^27^

In this work, zwitterionic sulfobetaine methacrylate (SBMA) is incorporated into the PDA matrix utilizing a one-step electropolymerization strategy. Analogous chemistry was applied for the synthesis of antifouling membranes^28^ and thermoresponsive hydrogels^29^ by means of oxidative polymerization. However, according to our best knowledge, the synthesis of hybrid PDA|PZ thin films by means of electropolymerization has not yet been reported. The platform electrodes for modifications are chemical vapor deposition (CVD)-synthesized boron-doped carbon nanowalls (BCNWs). They constitute a fractal structure^30^ of diamondized, vertically oriented sheets of graphene.^31^ The structural features of the bare BCNW and functionalized BCNW|PDA|PZ electrodes are investigated via scanning electron microscopy (SEM), Fourier transform infrared (FTIR) spectroscopy, and X-ray photoelectron spectroscopy (XPS) as well as nanomechanical testing. We have manifested the significant potency of BCNW electrodes in electrochemical sensing,^31−33^ attributed to the fast charge transfer kinetics and surface conductivity resulting from the interplay between sp^3^ and sp^2^ phases.

BCNWs can be thought of as an array of micro(nano) electrodes. There are several cases describing the electrochemical response depending on the topology of the diffusion fields. In particular, Case 3 is an array with partially overlapping diffusion fields and Case 4, with completely overlapping diffusion fields resulting in a response almost identical to that of an ideally planar electrode.^34,35^ Electrochemical investigations supported by density functional theory (DFT) computations show that pristine the BCNW exhibits Case 4 behavior, but application of the PDA|PZ coating shifts it to Case 3. Finally, we show that this phenomenon is responsible for enhancement of the electrode sensing properties in a neutral environment and in bovine serum solution.

## Results and Discussion

### Synthesis and Chemical Structure of PDA|PZ Coatings

The B:CNW growth mechanism is based on the joint chemistry of many species in plasma, not only limited to CN–, HyCNHx, BH–x, and CH+x radicals.^36^ Boron doping allows for the formation of sharp-edged, flat, and up to 3 μm long carbon walls^31^ with superior electrochemical performance (*k*^0^ = 1.1 × 10^–2^ cm s^–1^ and Δ*E* = 85 mV). On the contrary, undoped CNW samples do not present highly developed maze-like structures with high surface area impairing fast kinetics at the multiple redox centers (*k*^0^ = 3.7 × 10^–3^ cm s^–1^). Pristine CNWs revealed a significantly narrower electrochemical potential window with irreversible oxidation (+0.83 V) and reduction peaks (−0.50 V).^31^

**Figure 1:**
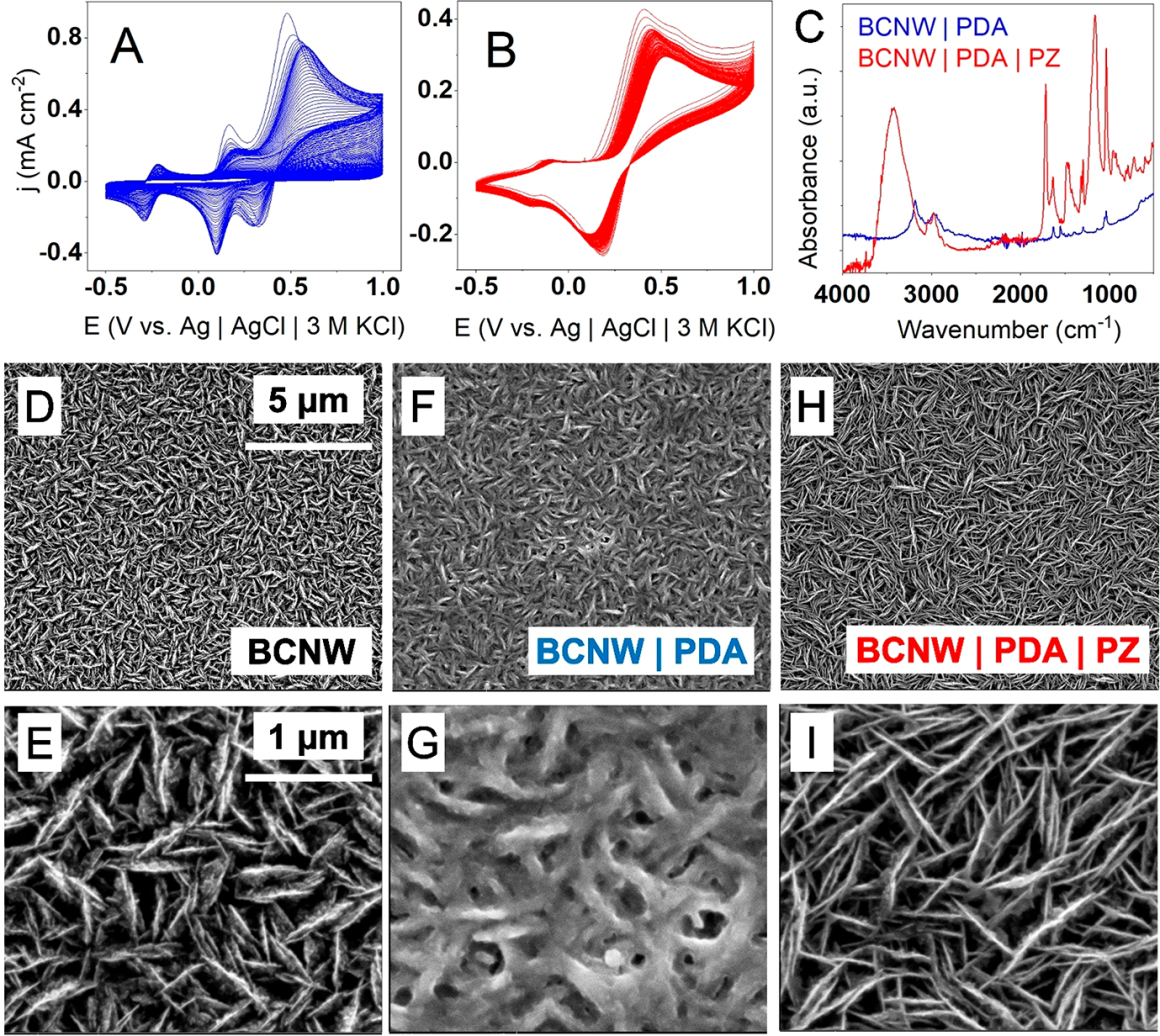
(A, B) Electropolymerization of polydopamine (PDA) and a hybrid PDA|PZ coating on boron-doped carbon nanowalls (BCNWs); (C) FTIR spectrum of deposited coatings; SEM pictures of BCNWs (D, E), BCNW|PDA (F, G), and BCNW|PDA|PZ, (H ,I) electrodes.

Both the PDA and PDA|PZ coatings were electropolymerized in argon-purged 1× Tris buffer (pH 7.4) containing 5 mM dopamine with or without an excess of 500 mM SBMA monomer. Significant differences between the deposition processes can be observed on cyclic voltammograms depending on the presence of the additional zwitterionic monomer (Figure 1A,B). In the case of sole dopamine solution, three quasi-reversible redox peaks are present, centered at −0.3, +0.1, and +0.5 V. Investigations from our previous work^37^ show that the peaks correspond to the redox couples as follows: the far cathodic peak to dihydroxyindole (DHI)–indole quinone (IQ), the middle one to leukodopaminechrome (LDC)–dopaminechrome (DC), and the last anodic one to dopamine (DA)–dopamine quinone (DQ), respectively. The current densities of each peak gradually decrease with consecutive cyclic voltammetry (CV) scans until very small currents (i.e., several μA) are reached. However, when zwitterionic monomer is introduced to the electrolyte (to a large extent, 500 mM), the deposition characteristics are altered. First, only two redox couples can be seen at −0.15 and +0.35 V, and second, they are shifted with respect to those in pure PDA deposition voltammograms.

Intermolecular electrostatic and π–cation interactions between PZ and PDA are responsible for the shift of the leftmost DHI/IQ peak toward more anodic potentials. Electrostatically positive N(CH_3_)_4_ groups can interact with the aromatic rings of the PDA, which leads to the withdrawal of the electron cloud from the ring.^38^ Therefore, further oxidation of DHI to IQ is energetically more favorable, and the peak shifts from −0.25 to −0.15 V. Partial justification of those phenomena are provided with the FT-IR spectra and ESP calculations (see further answers). It is foreseen that zwitterions could act catalytically for the DC isomerization to DHI. However, the detailed mechanism of this catalysis is unknown to us. Presumably, the intermolecular interaction between PDA and PZ leads to the stabilization of the quinone–methide intermediate, which occurs in living organisms (as described in ref (39)) and also is predicted from quantum chemical calculations^40^ (DFT B3LYP). This hypothesis would also explain the lack of the middle CV peaks corresponding to the LDC/DC pair in comparison to the pure PDA electropolymerization as all the DC would quickly isomerize to DHI.

Moreover, the current magnitudes are two times smaller but do not decay as fast with consecutive scans. This observation indicates that the hybrid coating is more conductive than PDA, which has the utmost importance in sensing applications. Higher conductivity could originate from the increased concentration of ionic charge carriers (generated via autoprotonation of dopamine or sulfonate groups^41^ or from an external solution) in the region between zwitterionic structural units.^42^

Additionally, a further study of the electrodeposition CV curves for varying PZ content was performed to verify the effect of PZ on the electropolymerization mechanism. At the lowest PZ content, they (Figure S2A,B) strongly resemble pristine PDA curves. In those cases, three pairs of redox peaks are present and the current gradually diminishes with consecutive cycling until 50 μA cm^–2^ is reached. However, for higher PZ contents starting from a 5:100 ratio (Figure S2C,D), currents do not plummet to very small values after 50 cycles. Simultaneously, the second oxidation peak corresponding to the LDC → DC reaction is smaller in comparison to others. It presumably originates from the higher contribution of π–cation interactions catalyzing chemical steps in the mechanism. Nevertheless, both CV curves and electrochemical impedance spectroscopy (EIS) spectra clearly indicate that, after deposition, the electrochemical activity is higher with increasing PZ content (Figure S2E,F). Low-rate CVs for the 5:100 PDA/PZ ratio show both oxidation and reduction peaks, but the oxidation peaks are flattened for higher rates. Moreover, Randles slope (*H*) rises with increasing PZ content, but the linearity is weaker for lower ratios. From those results, it can be inferred that the kinetics of charge transfer is generally slower at lower PZ contents. Therefore, choosing the highest possible PZ content limited by solubility (5:500) is optimal for maximizing the sensing performance of the material.

Insight into the structure of the PDA and PDA|PZ deposited on the BCNW can be obtained by the SEM inspection given in Figure 1D–I. Each picture displays vertically aligned graphite sheets confirming the structure of the nanowalls. Interestingly, the walls seem to be thicker after PDA deposition but thinner after hybrid coating. This second observation suggests that the incorporation of zwitterionic units into the polymer chains alters the interactions of PDA with the electron beam and ultimately the electrical properties of the surface.

The thickness of the coating was measured using AFM recorded at the edge of pristine and coated BCNW (Figure S3A–C). Then, several topography scans were conducted on the BCNW area of the specimen and on the BCNW|PDA or BCNW|PDA|PZ area. Each point on the histograms (Figure S3D,E) corresponds to the average of the single topography scan. When one calculates the average of those points for pristine and coated BCNW and subtracts the values, the thickness of the hybrid PDA|PZ coating could be obtained, and it is equal to 51 nm. The thickness of the PDA solely was equal to 6 nm; however, this value is in the experimental error and, therefore, gives only a rough approximation.

The chemical structure of the coatings was investigated by FTIR attenuated total reflection (ATR) spectroscopy (Figure 1C). While the signals of the BCNW|PDA electrodes are very weak and difficult to interpret, a spectrum of BCNW|PDA|PZ exhibits a well-pronounced series of absorption peaks. Considering the differences in signal intensities and clearness, one can anticipate that the zwitterionic units constitute a large portion of the hybrid coating structure. First, a broad medium band between 3200 and 3700 cm^–1^ corresponds to OH stretching vibrations of water adsorbed on the surface or absorbed into the structure.^43^ Hydroxyl groups of the PDA units also fall into this wavenumber range.^44^ Second, there are several small bands at ca. 3100–2800 cm^–1^ corresponding to the C–H stretching modes. They can originate from both aromatic (3100–3000 cm^–1^) and aliphatic (3000–2800 cm^–1^) C–H bonds of the PDA^44^ and SBMA units.^45^ Third, a band at 1720 cm^–1^ corresponds to C=O stretching vibrations of carbonyls in quinone-rich units of PDA^46^ or SBMA methacrylate parts.^45^ The low intensity band at 1650 cm^–1^ could correspond to C=C stretching modes in some indole rings in the PDA^47^ or unreacted adsorbed methacrylates.^48^ The small band at 1450 cm^–1^ is likely to be due to O–H deformation or C–N stretching vibrations.

The evidence supporting the idea of the zwitterion being incorporated into the coating includes two typical strong bands: a relatively broad one at 1160 cm^–1^ corresponding to S–O stretching and one at 1030 cm^–1^ related to C–O stretching modes.^45^ The signals in this range are significantly higher than in the case of pure PDA. The most probable origins of the variety of small bands in the range of 720–960 cm^–1^ are different modes of C–H groups such as bending, scissoring, and possibly C–S stretching.^49^ Those fingerprint bands occur only in the hybrid coating and therefore should come only from zwitterionic components of the coating. A similar set of bands, although slightly shifted, was observed in our previous work in a hybrid Nafion-co–PZ coating for glucose sensing applications.^50^

These bands are also visible on the sample of the pristine PZ coating (Figure S1). Generally, there are no substantial differences in the whole range of the spectrum except the C–H bond region (inset). Several C–H bands in the range between 2750 and 3150 cm^–1^ are diminished and shifted after deposition of the PZ. Considering that most of the C–H bonds are present in PZ, not PDA, these results suggest noncovalent π–cation interactions between tetraalkylammonium groups of PZ and aromatic rings of the PDA. Moreover, those changes support the fact that PDA undergoes cyclization to the indolequinone double-ringed units.

Another piece of evidence of the incorporation of zwitterions into the coating is the drastic decrease of the water contact angle from 123° for the pristine BCNW to 13° for the functionalized electrode. It is non-negotiable that the high level of acquired hydrophilicity must stem from surface modification with zwitterions.^10,12,43,51^

**Figure 2:**
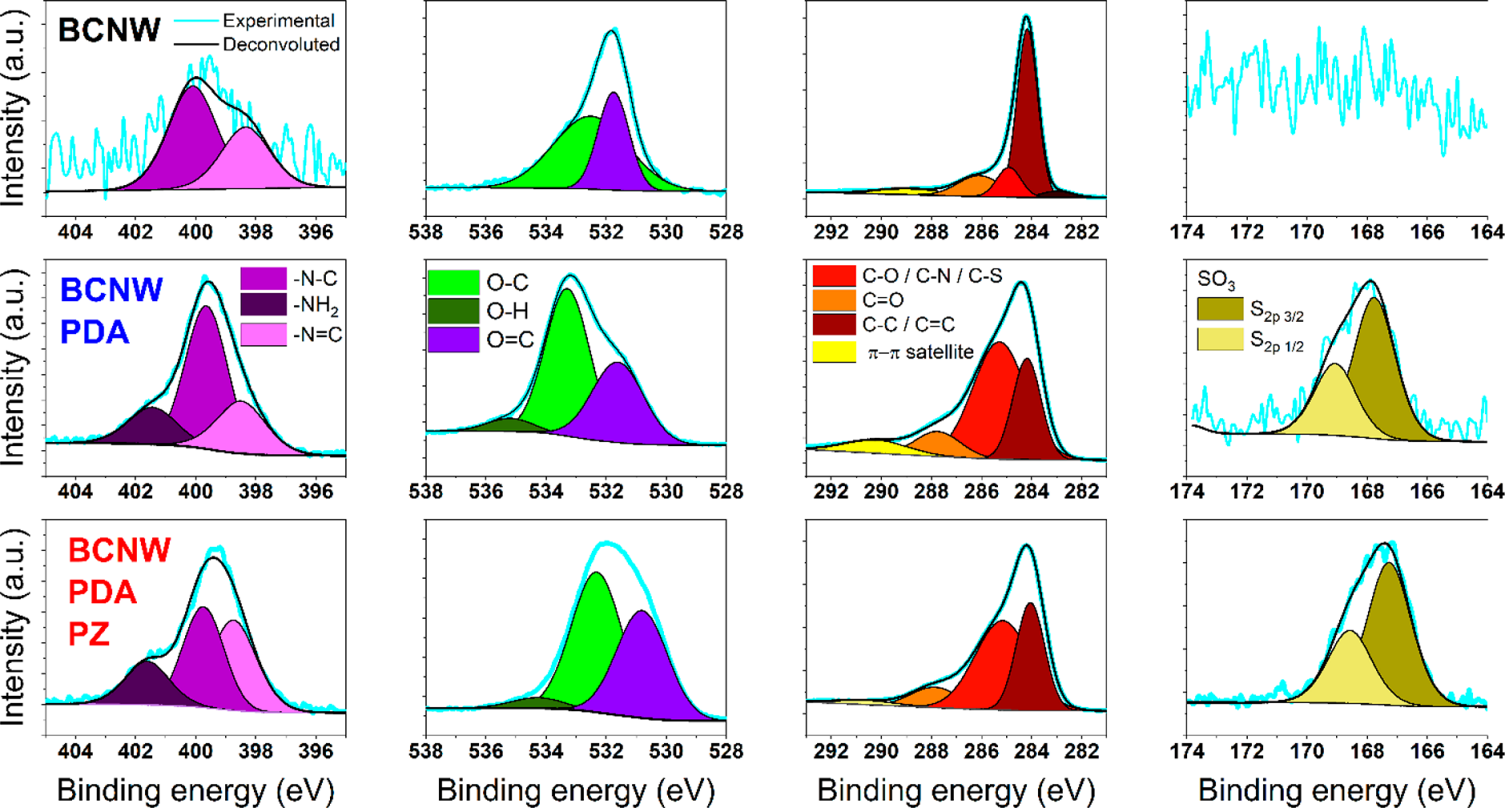
High resolution XPS spectra of BCNW, BCNW|PDA, and BCNW|PDA|PZ electrodes in the nitrogen, oxygen, carbon, and sulfur energy regions.

For further analysis of the chemical structure of the coatings, high resolution XPS spectra of bare and functionalized BCNW electrodes are provided in Figure 2. The nitrogen area of the spectra clearly shows that, in pristine BCNW, only trace amounts of nitrogen are present as a result of the 5% nitrogen in the plasma during CVD.^31^ They contribute to 0.57 atomic % in the form of N–C bonds at 400 eV and N=C bonds at 398 eV. However, after the deposition of the PDA or hybrid PDA|PZ coating, a notable increase was recorded, including the emergence of a third component at 401.5 eV, which was attributed to N–H bonds. Their presence comes from the primary and secondary amines commonly found in each structural unit of PDA. A similar set of peaks was observed in works in both freestanding PDA and PDA as a coating.^52−54^ In the oxygen region, the pristine BCNW spectrum consists of two deconvoluted components at 531.5 and 533 eV associated with C=O and C–O surface bonds, respectively. In our previous work, we showed that C–O bonds on the BCNW surface come in several variations, such as C–O–C and C–OH. The variety of C–O chemistries might explain the high fwhm of the C–O peak.^31^ After electropolymerization, an additional component is manifested at ca. 535 eV, originating from O–H bonds, presumably from residual water absorbed into the PDA and catechol groups. Narrowing of the C–O peak is a consequence of the fact that, in functionalized electrodes, the XPS signal originates from the C–O bonds of catechol and methacrylate groups rather than from the surface bonds of the BCNW that are far below the organic layer and do not interact with Ar+ ions during measurement. Finally, a slight increase of the C=O peak area and decrease of the O–H peak area can be observed for BCNW|PDA|PZ in comparison to that for BCNW|PDA. These two observations strongly suggest a higher oxidation state of the structural units in the hybrid coating compared to the pristine PDA.

Carbon parts of the XPS spectra reveal five components for bare BCNW and four components for functionalized BCNW. The differentiating peak is located at 283 eV and originates from C–B bonds. This signal vanishes after the modifications as a result of the finite penetration depth of the X-rays through the polymers. The four remaining peaks can be attributed to C–C bonds (284 eV), C–O and C–N bonds (285 eV), C=O bonds (287–288 eV), and a broad signal of π–π satellite distortions at 289–292 eV.^55,56^ It can be clearly seen that the C–O and C–N content rises after polymerizations, staying in agreement with the chemistry of PDA. Additionally, two deconvoluted peaks attributed to C–O and C=O shift from 285 to 286 eV and from 286.5 to 288 eV, respectively. The reason is that, in functionalized electrodes, the XPS signal is gathered from the incorporated C–O catechol and C–N amines, rather than from surface C–O–C bonds. Moreover, in the case of the BCNW|PDA|PZ, there is also a possible contribution of C–S bonds of the sulfobetaine unit in this range.^57−59^ In the hybrid coating, the signal corresponding to the π–π satellite peak plummets, presumably because SBMA units constitute a large portion of the coating. Since they have π bonds only in methacrylate groups, PZ does not contribute to the conjugated system of π bonds and the π–π signal is diminished.

The pristine BCNW does not contain sulfur compounds. However, XPS investigations show that PDA contains some number of sulfates, most probably originating from trace amounts of sulfates in the Tris buffer. In the case of PDA|PZ, those signals are amplified due to the presence of sulfonate groups in the SBMA monomers incorporated into the structure.

Several conclusions were drawn from the FTIR and XPS investigations. First, polydopamine is deposited onto the surface of the nanowalls, and SBMA zwitterions are incorporated into the PDA structure, most probably through noncovalent bonding involving π–cation interactions. A similar structure was proposed recently by Zhang et al.^29^ in the PDA|PSBMA composite, where PDA served as a noncovalent cross-linker. In the next section, nanomechanical studies are conducted to verify this hypothesis more rigorously.

### Nanomechanical Properties

**Figure 3:**
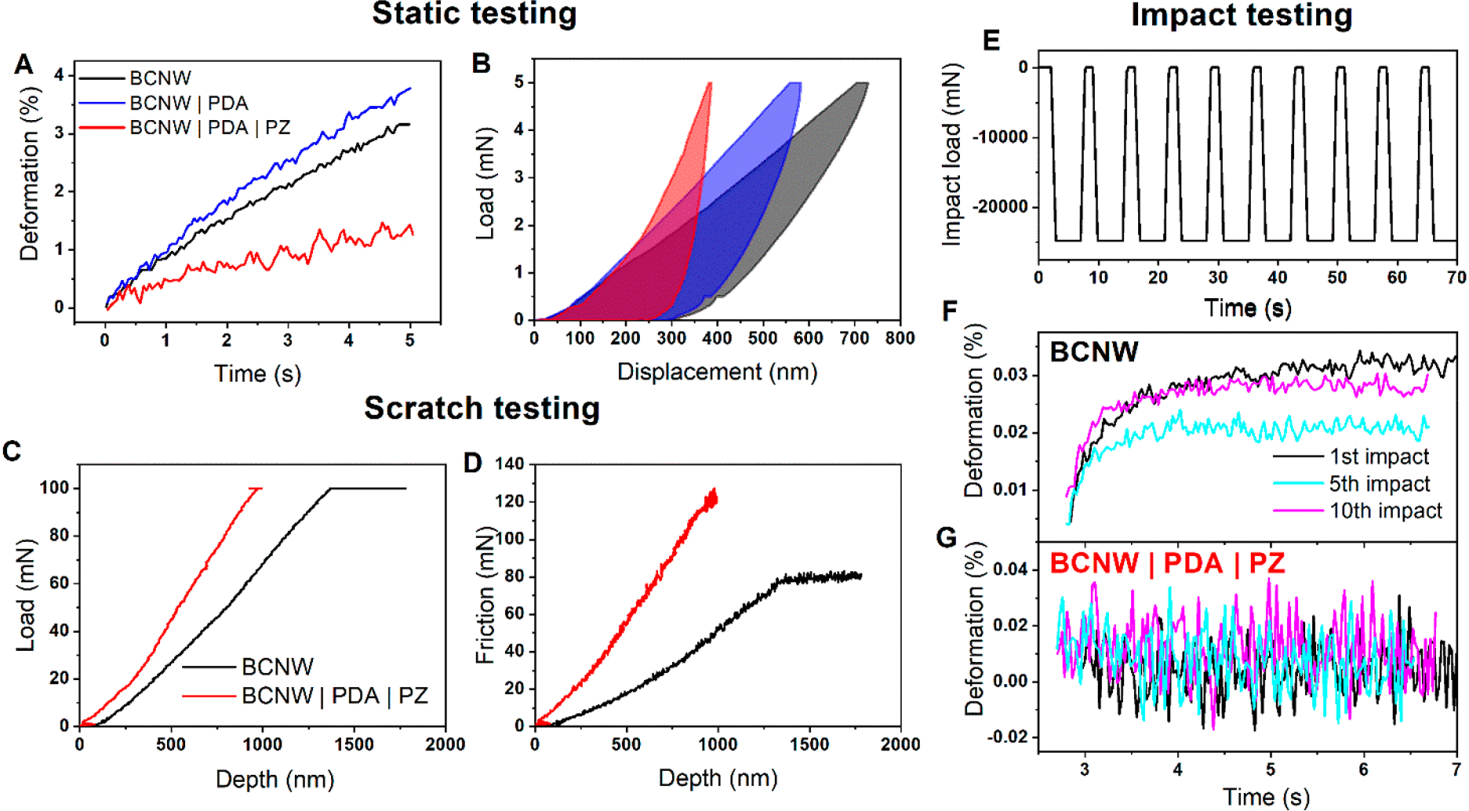
Nanoindentation studies of BCNW before and after electrodeposition of coatings. (A) Deformation velocity curves; (B) corresponding load–displacement curves; (C) load measured in the scratch test; (D) force of friction measured in the scratch test; (E) load profile applied during impact testing; (F) first, fifth, and 10th impact response of pristine BCNWs; (G) first, fifth, and 10th impact response of BCNWs functionalized with a hybrid PDA|PZ coating.

The AFM-estimated thickness of the PDA|PZ coating is equal to 51 nm and that of the PDA coating is only 6 nm. However, the second value is in the range of the experimental error and, therefore, is only approximate. For a better understanding of the relationships between the chemistry and surface properties of the functionalized BCNW, nanoindentation studies were performed in static, scratch, and impact modes (Figure 3). The load–displacement curves show that the pristine BCNW has the lowest stiffness and hardness, which increases after electropolymerization (see the calculated mechanical properties collected in Table S1). Pure PDA increases the Young’s modulus ca. 2 times, while hybrid PDA|PZ increases it up to 7.3 times. Moreover, the slope of the time–deformation curves is the lowest among the tested electrodes, indicating the increase of the rigidity of the nanowalls by PDA|PZ. Lastly, the final depth of the indenter in this experiment is the smallest, i.e., 376 nm in comparison to 698 nm for pristine BCNW, and the contribution of plastic deformation during the indent is the highest for the hybrid coating. These observations strongly suggest that the hybrid coating has more cross-links in the polydopamine backbone than PDA. They are presumably caused by electrostatic interactions between amphiphilic PZ molecules and π–cation interactions. It stands in agreement with the anticipated method of the incorporation of zwitterionic monomers into the coating during electropolymerization.^28^

The effect of increased hardness is also manifested in scratch tests (Figure 3C–D). For the plain BCNW, the desired 100 mN load is achieved at the depth ca. 1.35 μm, while in the case of BCNW|PDA|PZ, it is only 0.85 μm. Moreover, the friction force of the modified BCNW increases faster with the depth and reaches up to ca. 130 mN, which is almost two times higher with respect to the plain BCNW. Those observations indicate that modification with PDA|PZ reinforces the structure of the BCNW and increases the resistance against scratches.^60^ This feature is beneficial for biosensors working in fluid-flow conditions, where abrasion and wear through fluid friction could cause damage over time.

Impact testing reveals several important correlations between the mechanical properties and structure of the functionalized BCNW electrodes. The bare BCNW exhibits a swelling behavior after each impact, which is seen in Figure 3F as the large plateau of the deformation accompanied by smaller oscillations around the plateau. Additionally, after the first impact, the value of the plateau decreases from 0.03% to 0.02% at the fifth impact and the deformation velocity decreases to nearly zero, suggesting permanent distortion of the nanowalls. However, at the 10th impact, the plateau increases again and the deformation velocity is slightly larger than zero, which might be associated with relaxation through the fracture mechanism.^61^ A significantly different impact response is manifested by the functionalized BCNW (only BCNW|PDA|PZ is shown, but the response for BCNW|PDA is identical). Instead of the swelling, there are oscillations around the axis of zero deformation regardless of the number of impacts. The interpretation of this behavior is that after, coating the BCNW, the inner spaces between the walls are smaller or diminished completely so that there is no place for swelling. In other words, the nanowalls are forced to oscillate between their equilibrium positions because the coating changed the geometry and precluded the swelling. This observation provides further confirmation that PDA and PZ cause a profound alteration of BCNW surface properties, which is also revealed in the electrochemical performance.

### Electrochemical Properties

**Figure 4:**
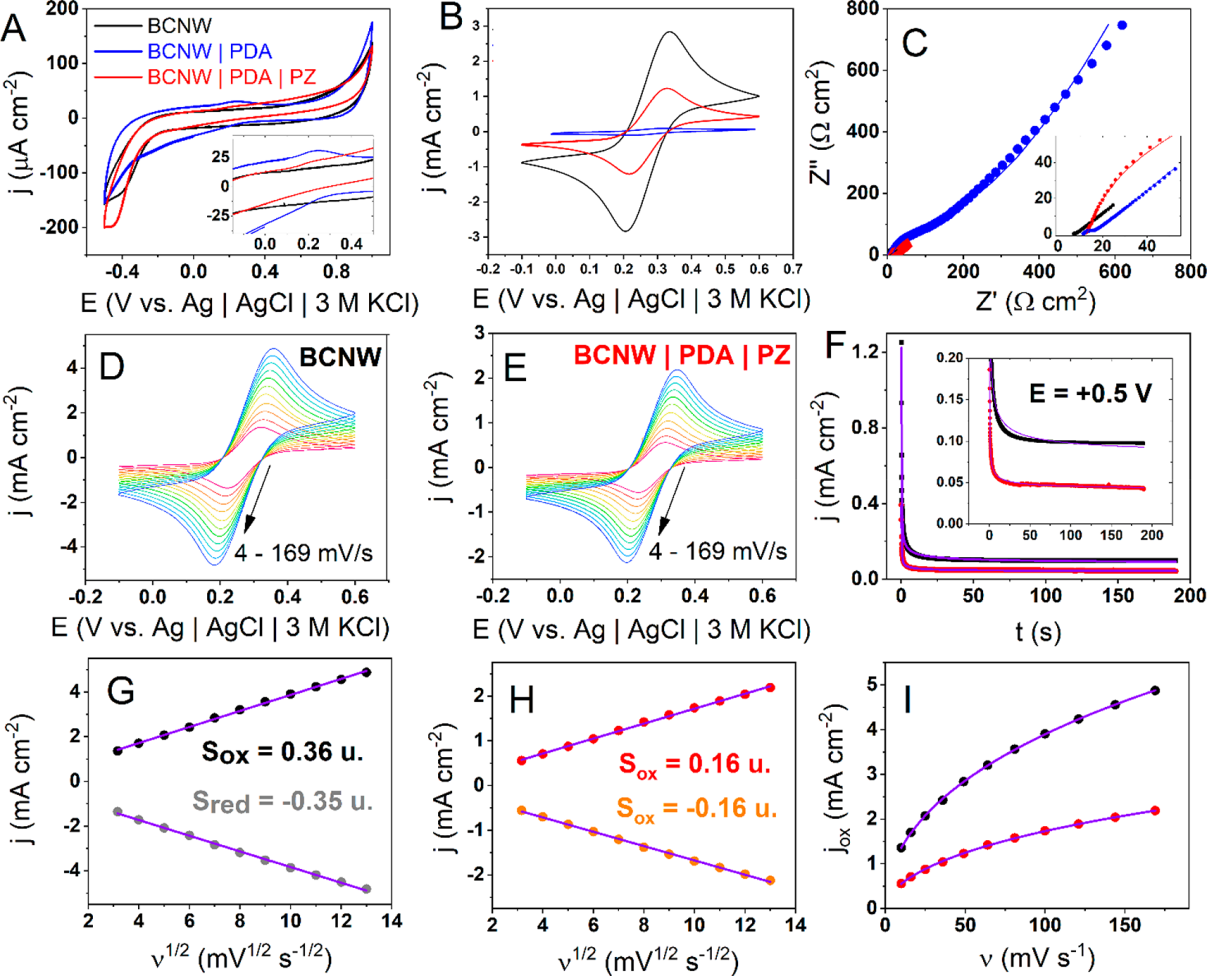
Electrochemical properties of pristine and functionalized BCNW electrodes. Comparison of CV curves recorded at 50 mV/s scanning speed (A) in 1× Tris buffer; (B) in 5 mM ferrocyanide solution; (C) EIS spectra (dots are experimental data, and lines are fitted data); (D, E) CV curves with different scan rates; (F) chronoamperograms registered at +0.5 V; (G, H) Randles-Ševčík plots, *S*_ox_ and *S*_red_ are slopes obtained through linear fitting and units (u) are mA cm^–2^ mV^–1/2^ s^1/2^; (I) nonlinear fitting of current–scan rate dependence; electrolyte for panels B–I was 5 mM K_3_Fe(CN)_6_ + 5 mM K_4_Fe(CN)_6_ + 1 M KCl; EIS spectra were fitted according the Randles circuit, i.e., *R*(*R*(*QW*)), *Q* is the constant phase element, and *W* is the Warburg element.

Cyclic voltammograms (CVs) of the pristine and functionalized BCNW electrodes in neutral (1× Tris) electrolyte are shown in Figure 4A. In the case of BCNWs, no significant currents from redox reactions can be observed in the window from −0.2 to +0.6 V; only capacitive currents (±20 μA cm^–2^) are present in this range. The PDA-functionalized electrode exhibits oxidation and reduction waves originating from redox reactions of catechol/quinone pairs that are abundant in most of the structural units of PDA.^16,62^ However, those currents are significantly smaller for the BCNW|PDA|PZ electrode. This observation stands in agreement with the FTIR and XPS investigations, because a large number of molecules in the PDA|PZ coating are zwitterionic units, not PDA, and they do not possess reactive catechol/quinone redox pairs.

Investigations of the processes occurring at the electrode/electrolyte interface were performed by measurements in a solution containing a 5 mM ferrocyanide redox pair. CV curves for pristine and functionalized BCNW electrodes are given in Figure 4B. For the unmodified BCNW, a pair of well-shaped, symmetric redox peaks with roughly 3 mA/cm^2^ current densities can be observed. The high electrochemical activity stems from the structure of nanowalls and was thoroughly studied in our previous works.^31,36^ Briefly, due to boron doping, they have high surface conductivity, and due to the interplay between the sp^2^ and sp^3^ phases, high charge transfer rates are achieved.

After electrodeposition of PDA solely, there is a significant loss in the redox response of ferrocyanides; current densities plummet to less than 0.1 mA/cm^2^, presumably due to adsorption of nonconductive products of dopamine oxidation. Interestingly, when a hybrid PDA|PZ coating is deposited, the decrease in the redox response of the ferroxyanides is smaller (40% of the bare BCNWs) and two redox peaks are preserved. This supports the hypothesis posed in previous sections that PDA|PZ results in higher surface conductivity than pure PDA.

A similar corollary can be drawn from the EIS measurements presented in Figure 4C and Table S2. In the case of pure PDA, both the real and imaginary impedances are significantly higher with respect to the pure BCNW. More precisely, there is a huge (80×) increase in charge transfer resistance *R*_ct_ and Warburg element magnitude *A*_w_ (14×), indicating the formation of a coating with low conductivity. However, in the case of the hybrid coating, the changes in impedance data are rather mild (3× increase of charge transfer resistance and 2× increase of the Warburg element magnitude), indicating the formation of the coating, but have less blocking than PDA solely. The kinetic constant *k*^0^ can be calculated on the basis of the charge transfer resistance from the equation:^63^
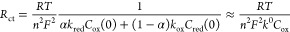
1where *R* is the gas constant, *T* is the temperature, *F* is the Faraday constant, *n* is the number of electrons involved in the reaction (*n* = 1), α is the charge transfer coefficient, *k*_red_ and *k*_ox_ are kinetic constants of the reduction and oxidation semireactions in the redox couple, and finally *C*_ox_ (0) and *C*_red_ (0) are the surface concentrations of the redox active species. The simplification is based on the fact that α ≈ 1/2, *C*_ox_ (0) ≈ *C*_red_ (0), and *k*_red_ ≈ *k*_ox_ due to the high degree of symmetry between oxidation and reduction. Similarly, the apparent diffusion coefficient *D* can be calculated from the value of the Warburg element according to eq 1:^63^
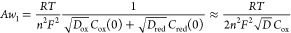
2where *D*_ox_ and *D*_red_ are diffusion constants of the oxidated and reduced agent of the redox (ferrocyanide) couple. The simplification is based again on the symmetry between reduction and oxidation mass transport so that *C*_ox_ (0) ≈ *C*_red_ (0) and *D*_ox_ ≈ *D*_red_ ≈ *D*. Lastly, the averaged double layer capacitance *C*_dl_ can be computed using the Brug formula (eq 3):^64^
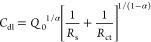
3where *R*_s_ is the solution resistance, *Q*_0_ is the magnitude of the CPE element, and *n* is the CPE exponent. It is easy to see that after deposition of PDA both the kinetic constant and diffusion coefficient plummet, but in the case of coelectropolymerization of PDA|PZ, the changes are moderate. Interestingly, the double layer capacitance is very similar for all three electrodes. Therefore, the modification does not influence this parameter of the electrode. When one considers that only the hybrid coating is more relevant from the sensing point of view, only this electrode will be subjected to further investigations.

CVs of the bare BCNW and BCNW|PDA|PZ recorded with different scan rates are shown in Figure 4D–E along with the Randles-Ševčík plots (Figure 4G–H). There is a high degree of symmetry between the oxidation and reduction currents in the whole range of scan rates, and the shapes of the quasi-reversible peaks are similar for both electrodes. However, the polymerized electrode currents are ca. 2 times lower in magnitude. Similarly, the Randles-Ševčík slopes are equal to 0.36 and 0.16 mA cm^–2^ mV^–1/2^ s^1/2^ for bare and coated BCNW, respectively, suggesting a roughly 50% reduction of the active surface area after electropolymerization. Interestingly, at the lowest scan rate, i.e., 9 mV/s, the peak-to-peak separation is equal to 90 mV for pristine BCNW and 80 mV for BCNW|PDA|PZ. In other words, the PDA|PZ coating leads to a mild decrease of both the apparent diffusion and kinetic constants (EIS results, Table S2), but the overall reversibility is increased. Nevertheless, it is still a mixed diffusion/kinetics-controlled process. The hypothesis explaining this behavior is as follows. The PDA|PZ coating decreases the active surface area of the electrode and induces specific changes in the surface geometry. As a result, the shape of the diffusion field around the nanowalls is shifted from heavily overlapped to moderately overlapped diffusion layers. In other words, the behavior of the BCNW after modification changes toward a microelectrode array, leading to the creation of a steady-state response.^34,35^

Several additional experiments were conducted to support this hypothesis. First, chronoamperometry measurements in the presence of 5 mM ferrocyanides were performed at the oxidative potential equal to +0.5 V so that the stationary state was obtained after about 1 min (Figure 4F). The resulting experimental curves for BCNW and BCNW|PDA|PZ were fitted using the modified version of the standard Cottrell equation^65^ (eq 4):
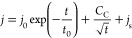
4where the exponential term describes the decay of current related to discharge of the double layer, *j*_0_ is a pre-exponential constant, *t*_0_ is the time constant of the capacitor discharge, and *C*_C_ is a Cottrell constant, proportional to the concentration of the electroactive species and the square root of the diffusion constant. For the purpose of this discussion, the most important is the last term, i.e., the steady-state current *j*_s_, which is the asymptotic current density when time approaches infinity. The physiochemical interpretation of this object is related to the geometry of the diffusion field in the vicinity of the electrode. In the case of a flat electrode, it equals zero, but for spherical or cylindrical electrodes, it is nonzero and dependent on the geometric parameters such as the electrode radius.^34,65^

Analogous behavior is observed for arrays of micro/nanoelectrodes and is captured by a theory of diffusive cases.^34,35^ If diffusion fields of electroactive antennas are separated, the steady-state current is nonzero. The greater the confinement of the fields, the higher the current amplification. This phenomenon is a valuable factor for electroanalysis because the steady-state current is more sensitive to trace amounts of analytes and independent of time.^35^ Simply, *j*_s_ is an indicator of the field geometry and can be used as a guide to distinguish between diffusion cases. Specific values of the parameters obtained through nonlinear fitting are gathered in Table 1. The curves are very well fitted with χ^2^ less than 3 × 10^–5^ and standard errors less than 2% each. There is one crucial difference between the pristine and functionalized BCNWs. Although the *j*_s_ value decreases after modification, the ratio between steady-state current *j*_s_ and the Cottrell current is greater and equal to 0.67 mA cm^–2^ s^–1/2^ compared to 0.44 mA cm^–2^ s^–1/2^ before modification. This almost 2-fold increase suggests that the contribution of the steady-state current to the overall current is higher in the case of the PDA|PZ functionalized electrode. Furthermore, the discharge time constant *t*_0_ is similar between the two electrodes, confirming the EIS results that the double layer capacitance is similar between the pristine and functionalized BCNWs.

**Table 1:** Resulting Values of the Nonlinear Fitting to the Experimental Chronoamperometric Curves According to Eq 4^a^

fitted parameter	BCNW	BCNW|PDA|PZ
*j*_0_ [mA cm^–2^]	1.64 (1.2%)	0.40 (1.0%)
*t*_0_ [s]	0.27 (1.1%)	0.29 (1.0%)
*C*_C_ [s^1/2^]	0.18 (0.5%)	0.06 (0.6%)
*j*_s_ [mA cm^–2^]	0.08 (0.3%)	0.04 (0.2%)
***j***_**s**_**/*C***_**C**_**[mA cm**^**–2**^**s**^**–1/2**^**]**	**0.44**	**0.67**
χ^2^ [10^–6^]	30.3	2.1

a Standard errors are given in brackets.

Another piece of information about the structure of diffusional fields can be drawn from the nonlinear fitting of the current density vs scan rate dependence (Figure 4I). Two functions were fitted according to the following eq 5:
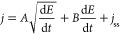
5where *A* is a constant related to the diffusive current proportional to the square root of the scan rate, *B* is a capacitive/adsorptive component of the current proportional to the linear scan rate, and finally *j*_ss_ is a measure of current independent of the scan rate. A similar equation is used to determine the capacitive and faradaic contribution in the fields of batteries and supercapacitors.^66−69^ The current is scan rate independent only when the steady state is reached, which happens in a rotating disk electrode experiment with a high angular frequency of rotations.^65^ Therefore, *j*_ss_ is another element indicating the contribution of the steady-state current to the overall current. The parameters obtained by nonlinear fitting are gathered in Table 2. It can be observed that *j*_ss_ is equal to 34 μA cm^–2^ for the pristine BCNW electrode and 63 μA cm^–2^ for the BCNW|PDA|PZ electrode, which is again almost two times the increase of the steady-state current after modification. An additional argument supporting the validity of this nonlinear fitting approach is the similarity between the estimated *A* values (0.43 and 0.20 mA cm^–2^ mV^–1/2^ s^1/2^) and the same values calculated via fitting according to the Randles-Ševčík formula (Figure 4G–H) (0.36 and 0.16 mA cm^–2^ mV^–1/2^ s^1/2^, respectively). The component *B* associated with the surface-bound reactions is close to zero and negligible in this case.

**Table 2:** Resulting Values of the Nonlinear Fitting to the Experimental Chronoamperometric Curves According to Eq 5^a^

fitted parameter	BCNW	BCNW|PDA|PZ
*j*_ss_ [mA cm^–2^]	0.034 (9.7%)	0.063 (37.2%)
*A* [mA cm^–2^ mV^–1/2^ s^1/2^]	0.43 (2.1%)	0.20 (3.0%)
*B* [mA cm^–2^ mV^–1^ s]	–0.0045 (12.4%)	–0.0020 (18.6%)
χ^2^	2.5 × 10^–4^	1.2 × 10^–4^

a Standard errors are given in brackets.

**Figure 5:**
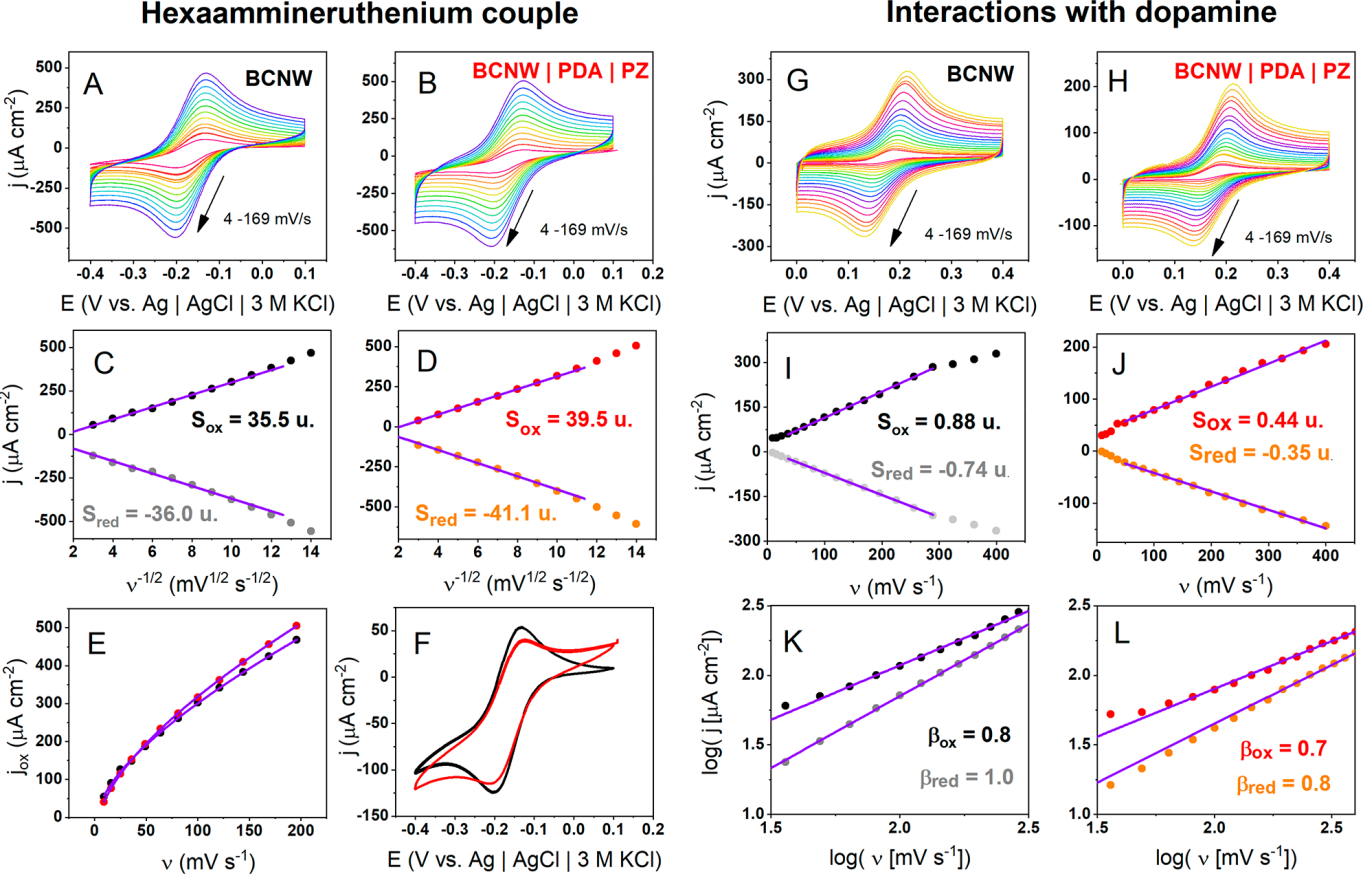
(A–F) Electrochemical properties of BCNW and BCNW|PDA|PZ in solutions containing a hexaammineruthenium redox pair and in dopamine: (A, B) CV curves with different scan rates; (C, D) Randles-Ševčík plots; units (u) of the slopes are μA cm^–2^ mV^–1/2^ s^1/2^; (E) nonlinear fitting of current–scan rate dependence; (F) comparison of CV curves for pristine and functionalized BCNW; (G–L) interactions of pristine and functionalized BCNW with dopamine; (G, H) CV curves with different scan rates; (I, J) linear current–scan rate dependencies; (K, L) log–log plots for current–scan rate dependencies.

Considering the above-discussed results, the hypothesis of alterating the diffusional field geometry by the PDA|PZ coating becomes an interesting idea, leading to several outcomes that may be fruitful in sensing applications. However, several additional experiments were performed to present more arguments supporting this approach. Electrochemical measurements of the pristine and functionalized BCNW electrodes in the presence of the hexaammineruthenium redox couple are presented in Figure 5A–F. Both electrodes exhibit two redox peaks, increasing with the scan rate. However, in contrast to ferrocyanide peak separations, they do not increase with the scan rate and are equal to 71 mV for BCNW and 65 mV for BCNW|PDA|PZ, which is a value very close to the theoretical 60 mV for an ideally reversible, purely diffusion-controlled system.^65^ This reduction of peak separation is attributed to the fact that the hexaammineruthenium couple has a lower reorganization energy than the ferrocyanide couple and therefore can be treated as an OSET (outer sphere electron transfer) redox pair.^70^ The Randles-Ševčík slope obtained from the oxidation peaks of the CV curves is equal to 35.5 μA cm^–2^ mV^–1/2^ s^1/2^ for pristine BCNW and 39.5 μA cm^–2^ mV^–1/2^ s^1/2^ for BCNW|PDA|PZ. Considering the almost pure diffusion control (in contrast to the mixed control of ferrocyanides), it is another suggestion that the surface of BCNW|PDA|PZ provides a more microelectrode-like geometry of diffusional fields for redox reactions, resulting in higher current densities. Asymmetries between the oxidation and reduction slopes can be attributed to the presence of nonzero background currents at the cathodic part of the potential window (see Figure 2A).

Analogous nonlinear fitting experiments for current vs scan rate dependence were also performed for the hexaammineruthenium pair (Figure 5E). In this case, there are no significant differences in the curves and in the fitting parameters. However, if we compare the cyclic voltammograms for the pristine and functionalized electrodes registered at very low scan rates (4 mV/s), an interesting difference can be seen. While the current density magnitudes are roughly the same for both, the BCNW|PDA|PZ electrodes exhibit a higher current after exceeding the peak potential, so that the overall CV shape is close to sigmoidal. When one considers the theory of microelectrode arrays and diffusion cases mentioned above, the microelectrode-like behavior of the BCNW|PDA|PZ is also suggested.

A similar methodology was applied to verify the interactions of dopamine with the surface of the pristine and the functionalized BCNW electrodes. Figure 5G,H shows CV curves registered at different scan rates. Two quasi-reversible redox peaks of the catechol/quinone redox couple are manifested, and their current density and peak separation increase with the scan rate. However, this reaction is surface-confined because this relationship is linear,^34,71^ in contrast to the quadratic relationship for classical redox couples (Figure 5I,J). There is also a 50% reduction in the slope (from 0.88 to 0.44 μA cm^–2^ mV^–1^ s) after modification, which is a similar behavior to the ferrocyanide couple. These observations strongly suggest that dopamine–dopamine quinone redox reactions are inner-sphere electron transfer (ISET), confirming the well-described mechanism.^17,72^ Lastly, log–log plots of the current density vs scan rate were calculated according to eq 6:^68,73^
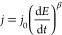
6Exponent β is an alternative tool for estimating the contributions of different types of current (capacitive/absorptive, diffusive, and steady state). In the case of bare BCNW, it is equal to 0.8 for oxidation and 1.0 for reduction, indicating that there is a primary capacitive/adsorptive component. Values close to 1.0 explain the linear dependence observed in Figure 5I,J. However, for BCNW|PDA|PZ, there is a notable decrease in the exponents for both oxidation (0.7) and reduction (0.8). Considering that the exponent for the processes in the steady state is equal to zero, the lowered value for the functionalized electrode is another clue suggesting shifts in the diffusional fields toward microelectrode-like behavior.

### Influence of Diffusion Field Shift on the Sensing Properties of BCNW|PDA|PZ

Considering a high potency of BCNW toward sensing and the specific electrochemical properties acquired after modification with PDA|PZ, a set of dopamine sensing experiments was conducted. This particular choice of analyte was motivated by the fact that the polydopamine moieties linked to the carbon surface could increase the selectivity via the creation of hydrogen and electrostatic interactions with a chemically similar analyte, dopamine. This mechanism is commonly utilized for molecular imprinting applications with PDA as the template,^25^ e.g., in the detection of proteins,^74^ nitroaromatic explosives^75^ and insecticides.^76^

**Figure 6:**
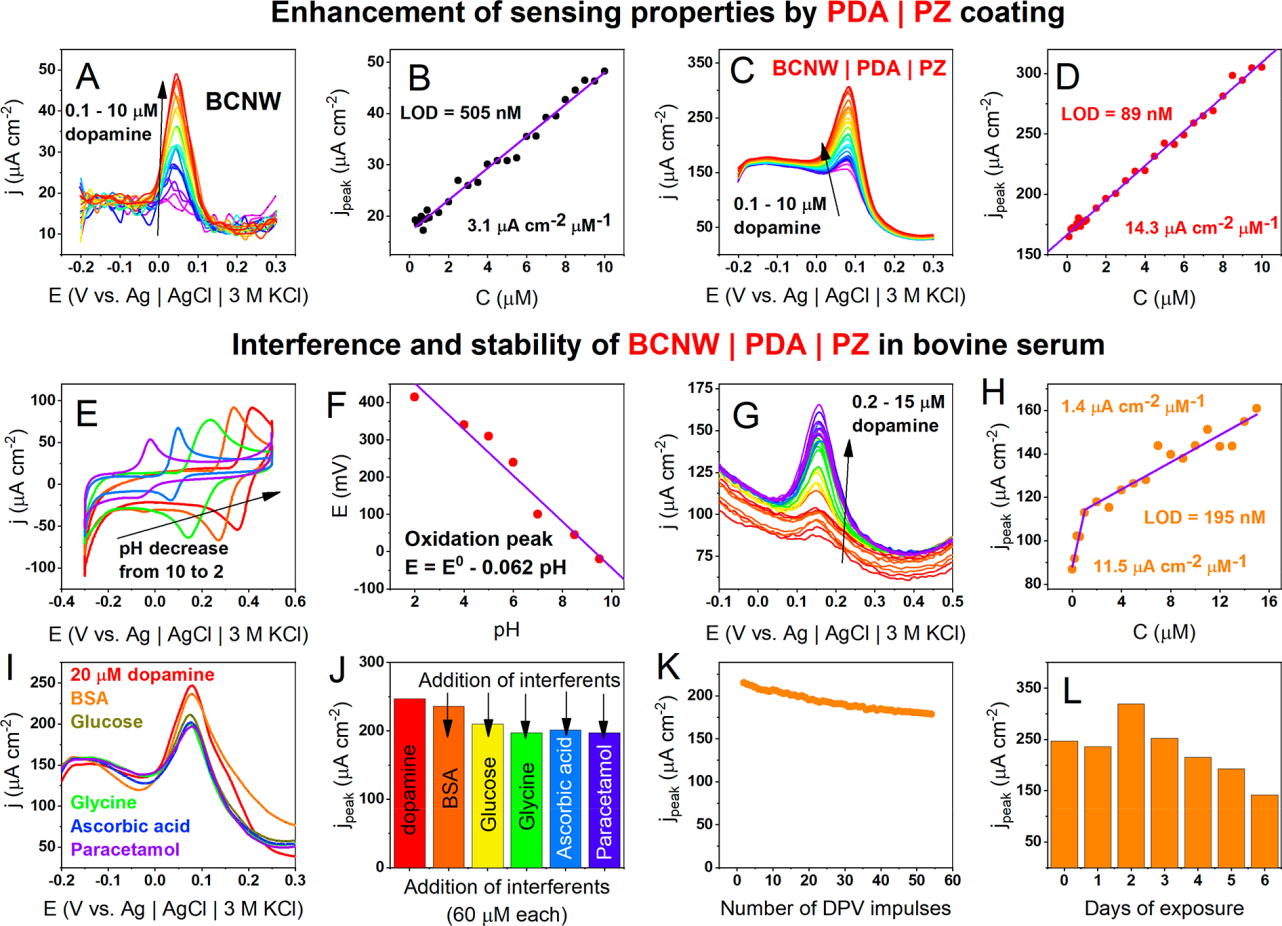
(A–D) DPV current response of pristine BCNW and BCNW|PDA|PZ in increasing concentrations of dopamine; (A, C) DPV curves in 1× Tris; (B, D) calibration curves in 1× Tris; (E–L) sensing properties of BCNW|PDA|PZ in bovine serum; (E) dopamine oxidation in different pHs; (F) dependence of oxidation potential peak on the pH; (G) DPV curve in BSA; (H) calibration curves in BSA; (I) DPV curves in BSA in the presence of interferents (red, dopamine; orange, BSA, green–yellow, glucose; green, glycine; blue, ascorbic acid; violet, paracetamol); (J) bar plot of current densities in the presence of interferents; (K) stability of the response in the subsequent 55 DPV cycles in BSA; (L) long-term stability test in BSA.

Difference pulse voltammetry was used for dopamine detection. Figure 6A–D shows a comparison of the sensing properties between the plain and PDA|PZ functionalized BCNW in 1× Tris buffer solution. In the case of both electrodes, a set of characteristic current pulses is observed with magnitudes that linearly increase with the dopamine concentration. Although the CV measurements showed dopamine oxidation onset at +0.17 V, the peaks on the DPV curves are located at +0.05 V, which is a standard behavior.^65^ However, while a pristine BCNW electrode exhibits a symmetric current pulse, BCNW|PDA|PZ increased the current plateau at potentials lower than the pulse. In other words, zwitterionic modification reduces the onset of the dopamine oxidation potential. Simultaneously, the slope of the calibration curve is 4.6 times higher, while the limit of detection decreases from 505 to 89 nM. Moreover, the linear range is slightly widened in both lower and higher conentrations (see Table 3). A comparison of those values with several recently published dopamine-sensitive materials leads to the conclusion that the BCNW|PDA|PZ electrode exhibits superior sensitivity while maintaining a solid compromise with the limit of detection (LOD) and linear range. The improvement of the three described sensing parameters is explained by the fact that the application of a hybrid PDA|PZ coating on BCNW leads to shifts in the diffusional fields toward more microelectrode-like behavior. Due to the higher contribution of the steady-state current, the increase of the current response is attributed to dopamine oxidation and an amplification of the signal at lower concentrations is observed. Furthermore, zwitterions are known to improve the transport of different charged molecules by forming ionic transport channels.^77−79^ It is therefore anticipated that surface zwitterions in BCNW|PDA|PZ play a supporting role in the enhancement of dopamine sensing. This issue will be revisited in the next section, including theoretical DFT investigations of zwitterion–dopamine intermolecular interactions.

**Table 3:** Comparison of the Sensing Properties of Different Electrodes toward Dopamine Electrooxidation in Neutral Media

material	electrochemical method/solution pH	sensitivity [μA cm^–2^ μM^–1^]	linear range [μM]	LOD [nM]	reference
CdSe/CdS quantum dots on graphite	DPV/pH = 11	0.05	0.5–15	96	(80)
N-doped porous carbon	DPV/pH unknown	4.64	30–90	2.74	(81)
			200–400		
CNTs@nanoporous carbon	DPV/pH = 3.0		0.5–49	20	(82)
Ni-MOF/glassy carbon	DPV/pH = 7.2	0.07	2–10	60	(83)
laser-induced graphene from biomass	DPV/pH = 7.2	4.39	5–40	3400	(84)
bioassisted AgNP	DPV/pH = 7.2	0.27	10–100	100	(85)
AgNPs/CuO	CV/pH = 7.5	3.00	0.04–10	7	(86)
aptamer IMIP/AuNPS/GC	DPV/pH = 7.2	0.7	0.05–10	47	(87)
Fe3O4@SiO2–laccase-GCE	DPV/pH = 6.0	0.23	1.5–75	177	(88)
exopolysaccharide/laccase/MWCNTs/GCE	SWV/pH = 6.0	0.58	3–38.5	127	(89)
MoS2–laccase@Nafion–carbon paper	CA/pH = 6.0	0.34	0.1–0.5	10	(90)
DNA/PAMAM/MWCNT-chitosan/Au	DPV/pH = 7.4	0.3	0.2–10	30	(91)
polypyrrole/MWCNT/GCE	DPV/pH = 7.0	0.09	0.6–100	60	(92)
**BCNW**	**DPV/pH = 7.4**	**3.1**	**1–10**	**505**	**this work**
**BCNW|PDA|PZ**	**DPV/pH = 7.4**	**14.3**	**0.7–20**	**89**	**this work**

The sensing of dopamine on the BCNW|PDA|PZ electrodes is possible in a wide pH range from 2 to 10 (Figure 6E). In more alkaline solutions, the amperometric signal is quenched, presumably because of the adsorption of different oxidation products, leading to the loss of PDA|PZ conductivity. Furthermore, the dependence of the reaction potential on the pH exhibits slopes equal to 0.062 V/pH units (close to the theoretical 0.059 V/pH units), indicating that the dopamine oxidation is proton-coupled (Figure 6F).

Further investigations include dopamine sensing in an environment reflecting body fluid conditions, particularly in bovine serum with a high albumin concentration (2 wt %). Dopamine oxidation DPV peaks are still present and increase with the concentration of dopamine. However, the prepeak current plateau plummets in contrast to that of the 1× Tris buffer solution, and the calibration curve has a different shape (Figure 6H). Two linear ranges of 200–1000 nM and 1–15 μM are present with 11.5 and 1.4 μA cm^–2^ μM^–1^ sensitivities, respectively. The first sensitivity value is very close to the slope measured in 1× Tris. This behavior suggests a transition to a different sensing mechanism,^93^ presumably due to interactions of dopamine with proteins. In higher dopamine concentrations, it can spontaneously bind to tryptophan and tyrosine residues of albumins by hydrogen bonding (DA–albumin).^94,95^ As a result, the chemical moiety being sensed in BSA is not pure DA but DA–albumin. According to the standard theory of voltammetry, the current–concentration slope depends on the electroactive surface area, diffusion coefficients, and kinetic parameters.^65^ Therefore, the sensitivity decreases with higher dopamine concentrations either due to blocking of the surface by DA–albumin or due to transport inhibition by the albumin adjoint. Regardless of the mechanism change, at a lower concentration range, the limit of detection remains very low, 195 nM.

Dopamine sensing was tested for interference by several other electroactive, physiologically abundant compounds such as glucose, glycine, ascorbic acid, and paracetamol. Interferents were subsequently added to the electrolyte in 3 times higher amounts than dopamine, i.e., 60 μM. Despite the significant excess of interferent, dopamine oxidation pulses were observed (Figure 6I) in a similar potential range. The current density dropped (16%) after the addition of glucose, although the other interferents seemed to not significantly influence the dopamine signal. Additionally, two stability tests were performed in BSA solution. The first included 55 subsequent DPV pulses (Figure 6K), and the second included a continuous exposure to BSA containing dopamine and interferents for a week (Figure 6L). After 55 pulses, the current density at the peak decreased (17%), but the overall sensing capability was not altered. Long-term exposure showed that the amperometric response was maintained for 5 days (254 ± 39 μA cm^–2^) and started to decrease after the sixth day. This behavior can be ascribed to the protective role of zwitterion preventing the adsorption of albumins.^8,12,59^

### Intermolecular Interaction between Zwitterion and Dopamine

**Figure 7:**
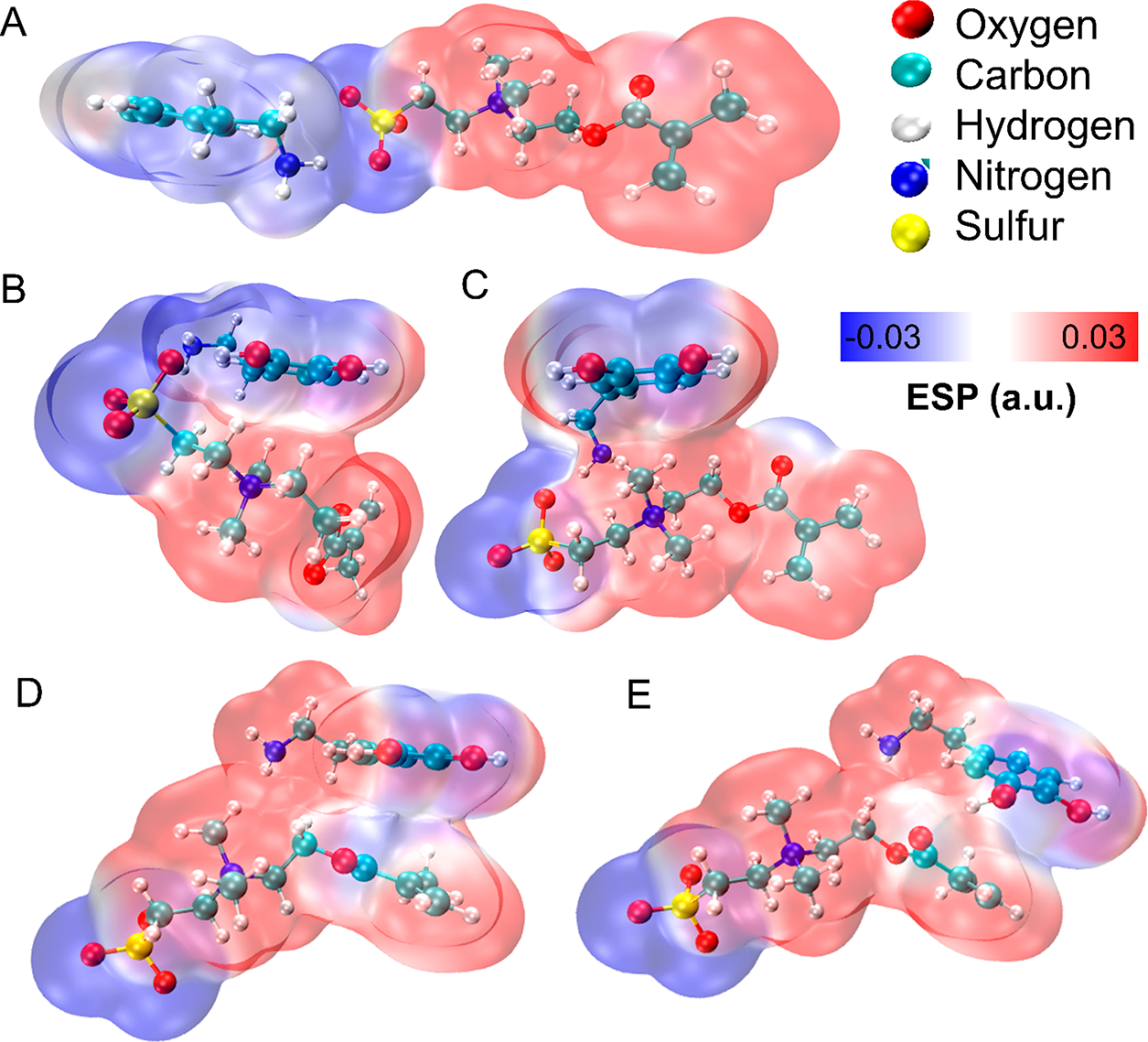
Simulation of intramolecular mechanisms underlying enhancement of the BCNW|PDA|PZ sensing capabilities. (A–E) Series of optimized geometries and electrostatic surface potential maps illustrating the intermolecular interactions between dopamine that facilitate transport of the analyte toward the surface.

This section is devoted to the elucidation of the intermolecular interactions between the zwitterion (SBMA) incorporated into the PDA structure and the analyte, dopamine (DA). Particular focus is put into finding the molecular mechanism explaining the experimentally observed shifts in the diffusion fields after application of the PDA|PZ coating. Therefore, a series of geometry optimizations was performed for different spatial arrangements along the zwitterion molecule and electrostatic surface potential maps (ESPs) are plotted for each step. The idea was to simulate the transport of dopamine analyte toward the electrode in the presence of zwitterion (Figure 7).

The initial contact (Figure 7A) of the two molecules starts by forming a bond of 3.14 Å length between the primary amine of DA and the sulfonate group of SBMA. The next step involves a rigid rotation of dopamine accompanied by breaking of the sulfonate–amine bond and creation of the π–cation interaction between tertiary amine and the aromatic ring (Figure 7B). Alternatively, dopamine can undergo a rotation along the alkyl chain also resulting in π–cation interactions (Figure 7C). The distance between the methyl carbon and the phenyl ring is equal to 3.61 Å, indicating a relatively strong affinity toward these groups.^96^ Either way, a translation toward the polyzwitterion alkyl chain is thermodynamically favored.

The next step of the dopamine transport is breaking of the as-formed bonds and forming the π–π interaction between the phenyl ring and the π electrons of the methacrylate group (Figure 7D). This distance is equal to 3.68 Å, which indicates a very strong π–π interaction; however, the changes of the ESP values are rather mild.^97^ At the last step, as the dopamine is translated further, another hydrogen bond is formed between the catechol and methacrylate oxygens with a length of 2.84 Å (Figure 7E).

**Figure 8:**
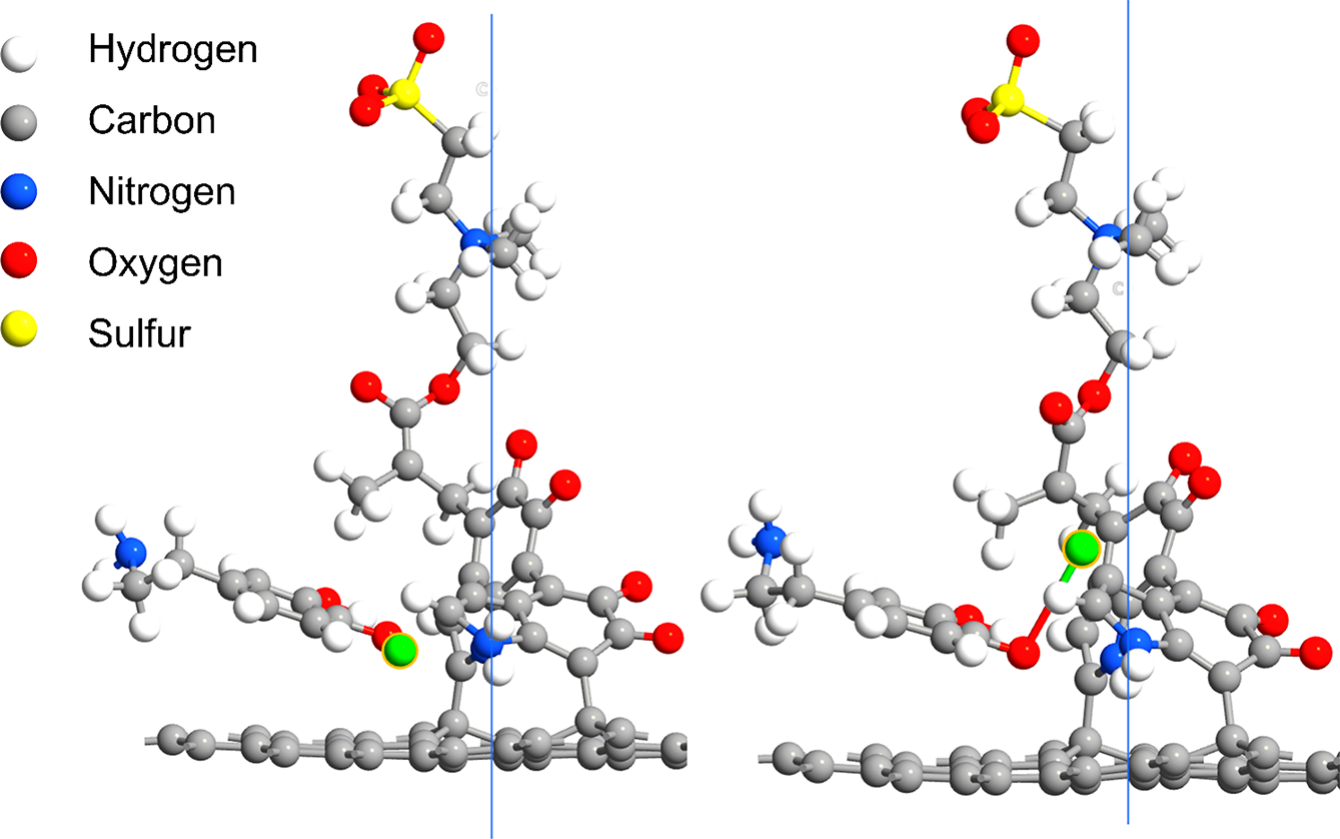
Left panel: Dreiding preoptimized geometry of the dopamine adsorbed on the carbon surface coated with PDA|PZ and formation of the molecular pocket for dopamine; Right panel: DFT full optimization of the adsorbed dopamine and geometry illustrating the stabilization of the transition state in the dopamine–dopamine quinone oxidation reaction; the green atom is hydrogen abstracted from the catechol group.

Finally, dopamine is adsorbed onto the surface of the BCNW coated with PDA|PZ. Results of preoptimization using the Dreiding force field show that polydopamine units attached to the carbon surface create a specific environment for the dopamine–analyte consisting of several hydrogen bonds and π–π interactions (Figure 8, left panel). In this geometry, a molecular host for dopamine oxidation is formed. In fact, this phenomenon is similar to the molecular imprinting studies, where PDA serves as a template.^25,75,76,87^ Instead of imprinting a protein or nitroaromatic explosive for detection in a PDA matrix, the PDA itself is the template for dopamine sensing. Another corollary confirming this statement can be drawn from analysis of the DFT-optimized molecular pocket (Figure 8, right panel). After full optimization, a hydrogen atom from the catechol group (marked as green) is abstracted from the dopamine and transported toward one of the DHI units of polydopamine.

In the standard dopamine electrooxidation mechanism utilized for sensing,^83−85^ dopamine gets oxidized to the dopamine quinone, and this reaction is coupled with the abstraction of two protons (see Figure 6E). The following DFT calculations strongly suggest that the transition state of this reaction (i.e., the moiety with only one abstracted hydrogen atom) is stabilized by intermolecular interactions induced by the pocket. In other words, dopamine oxidation is thermodynamically easier when electropolymerized PDA is present on the surface. It is believed that both transport facilitation by PZ and the formation of the molecular host by PDA explain the above-mentioned enhancement of the dopamine sensing parameters of the BCNW|PDA|PZ electrode.

## Conclusions

In summary, zwitterions can be incorporated in the structure of PDA by coelectropolymerization with dopamine on the BCNW surface. FTIR and XPS studies suggest that it most probably occurs through noncovalent electrostatic and π–cation interactions, and it leads to a higher physical cross-linking density than in pure polydopamine. Nanoindentation studies show that the hybrid coating exhibits enhanced stiffness, hardness, and the resistance of BCNW against scratches, reflected by a higher cross-linking density.

The functionalized BCNW electrode has promising capabilities for the detection of dopamine. Although the application of pure PDA causes quenching of the electron transfer, the hybrid coating only mildly changes the kinetic constant, and the high electrochemical activity of the BCNW substrate is maintained. In particular, BCNW|PDA|PZ exhibits up to 5 times the enhancement of sensitivity and 5 times reduced LOD after modification with PDA|PZ. This improvement is ascribed to the shifts of diffusional fields toward more microelectrode-like behavior. A large set of electrochemical experiments, directed toward examination of steady-state currents using different redox mediators and polarization conditions, support this hypothesis. Moreover, DFT calculations showed that zwitterions facilitate the transport of dopamine to the electrode via a chain of subsequent intermolecular interactions. It is composed of several hydrogen bonds and cation−π and π–π interactions. The PDA anchored to the surface, on the other hand, creates an environment that stabilizes the transition state between the dopamine and dopamine quinone, a molecular pocket. These phenomena are believed to be another mechanism explaining the enhanced sensing parameters. Additionally, zwitterions protect the electrode against interference and support the stability of the signal during several days of continuous exposure to the albumins in bovine serum.

## Methods

### Boron-Doped Carbon Nanowall (BCNW) Fabrication

BCNW electrodes were manufactured on p-type (100) Si wafer plates utilizing a microwave plasma-enhanced chemical vapor deposition system (SEKI Technotron AX5400S, Japan) as reported elsewhere.^98^ The stage temperature was set to 700 °C, while the microwave power was kept at 1300 W. The fabrication process was carried out at a pressure of 50 Torr in a gas mixture flow equal to 325 sccm. The CNW electrodes were boron-doped in situ utilizing a diborane precursor ([B]/[C] ratio in the gas phase = 2000 ppm). The 6 h process resulted in thick vertically aligned carbon nanowall surfaces (*d* ∼ 3 μm) with a high content of the diamond phase.^30^

### Electropolymerization of Dopamine and PZ Coelectropolymerization

50 voltammetry cycles were used for all electropolymerization experiments with a 20 mV/s scan rate and at the potential range from −0.5 to +1.0 V vs. Ag|AgCl|3 M KCl. In the case of pure PDA deposition, the solution consisted of 1× Tris and 5 mM dopamine and was purged with argon 15–20 min before starting the deposition. In the case of coelectropolymerization, 500 mM SBMA monomer was also added. The pH for deposition was adjusted to 7.2 by adding 1 M NaOH. An MP-103 hand-held potentiometric pH meter was used to control the pH value.

## Supporting Information Available

Figure S1, the comparison of FT-IR spectra registered for pure PZ and BCNW coated with PDA|PZ; Figure S2, electropolymerization CV curves and electrochemical studies for PDA|PZ deposited with variable PDA/PZ ratios in the electrolyte; Figure S3, AFM images of BCNW, BCNW|PDA, and BCNW|PDA|PZ with histograms used for estimation of the coating thickness; Tables S1 and S2, detailed values of the nanomechanical properties and physiochemical quantities obtained via nonlinear fitting of EIS data, respectively; more thorough description of the methods used throughout the manuscript: data on reagents, SEM inspection, XPS and FT-IR spectroscopic measurements, nanoindentation methodology, electrochemical characterization, and DFT computational details (PDF)
