# Supplementary material for: Tailoring Diffusional Fields in Zwitterion/Dopamine Copolymer Electropolymerized at Carbon Nanowalls for Sensitive Recognition of Neurotransmitters
Source: ACS Nano. 2022 Jul 22;16(8):13183–98. doi: 10.1021/acsnano.2c06406 (PMC9413423; doi:10.1021/acsnano.2c06406)
Supplement: Supplementary file 1 — nn2c06406_si_001.pdf [file nn2c06406_si_001.pdf]

Tailoring diffusional fields in zwitterion/dopamine copolymer electropolymerized at carbon nanowalls for sensitive recognition of neurotransmitters

Adrian Olejnik,[1,2] Mateusz Ficek,[1] Marek Szkodo,[3] Alicja Stanislawska[3], Jakub Karczewski,[4] Jacek Ryl,[4] Anna Dołęga,[5] Katarzyna Siuzdak[2] and *Robert Bogdanowicz[1]

[1]*Department of Metrology and Optoelectronics, Faculty of Electronics, Telecommunications and Informatics, Gdańsk University of Technology, Narutowicza 11/12 St., 80-233 Gdańsk, Poland Narutowicza 11/12 St., 80-233 Gdańsk, Poland*

[2]*Centre for Plasma and Laser Engineering, The Szewalski Institute of Fluid-Flow Machinery, Polish Academy of Sciences, Fiszera 14 St., 80-231 Gdańsk, Poland*

[3]*Department of Materials Engineering and Bonding, Faculty of Mechanical Engineering and Ship Technology, Gdańsk University of Technology, Narutowicza 11/12 St., 80-233 Gdańsk, Poland Narutowicza 11/12 St., 80-233 Gdańsk, Poland*

[4]*Institute of Nanotechnology and Materials Engineering and Advanced Materials Center, Gdańsk University of Technology, Narutowicza 11/12, 80-233 Gdansk, Poland*

[5]*Department of Inorganic Chemistry, Faculty of Chemistry, Gdańsk University of Technology, Narutowicza 11/12 St., 80-233 Gdańsk, Poland*

***Corresponding author:** E-mail: rbogdan@eti.pg.edu.pl. Tel: +48-58-347-15-03. Fax: +48 58-347-18-48 (Robert Bogdanowicz)

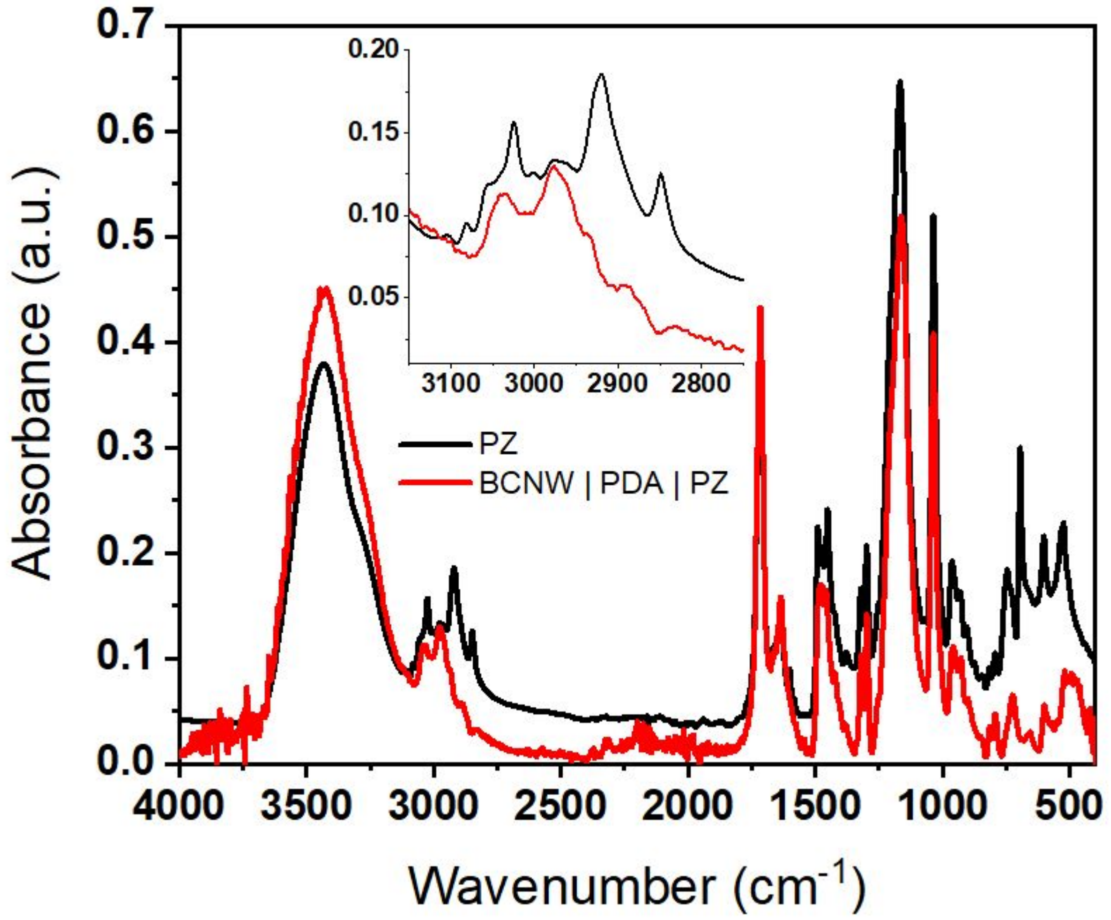

Figure S1. Comparison of FT-IR spectra registered for pure PZ and BCNW coated with PDA | PZ

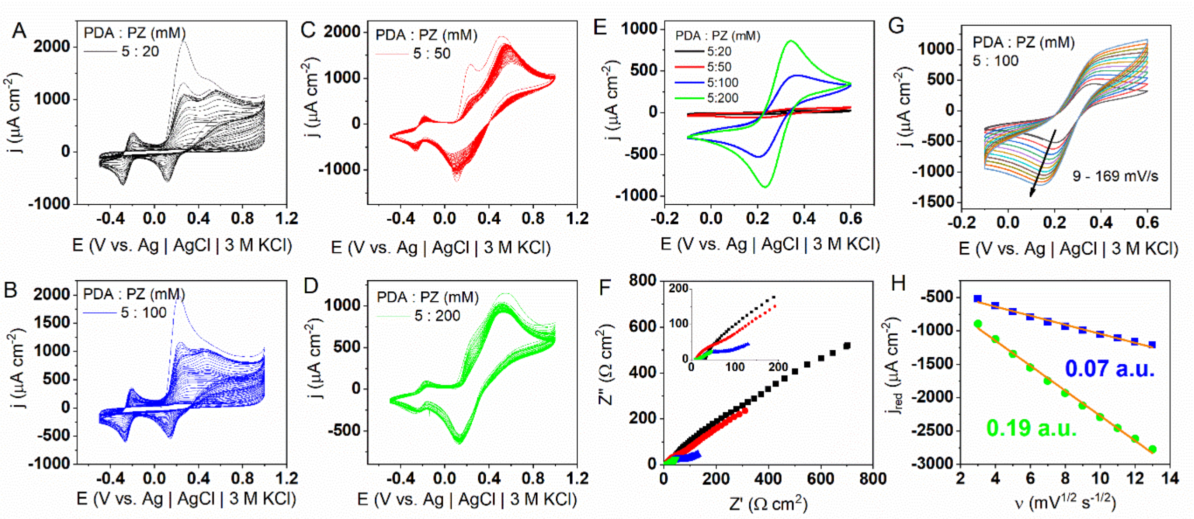

Figure S2. A-D - Electropolymerization CV curves for PDA | PZ deposition with variable PDA : PZ ratio in the electrolyte, 5 X Tris buffer was used (pH = 7.2); E-F - comparison of CV curves and EIS spectra registered for BCNW | PDA | PZ with different PDA : PZ ratio; G – CV curves with varying scan rates for BCNW | PDA | PZ with 5 : 100 ratio; H – Randles-Sevcik plots for 5:100 and 5:200 PDA : PZ ratios

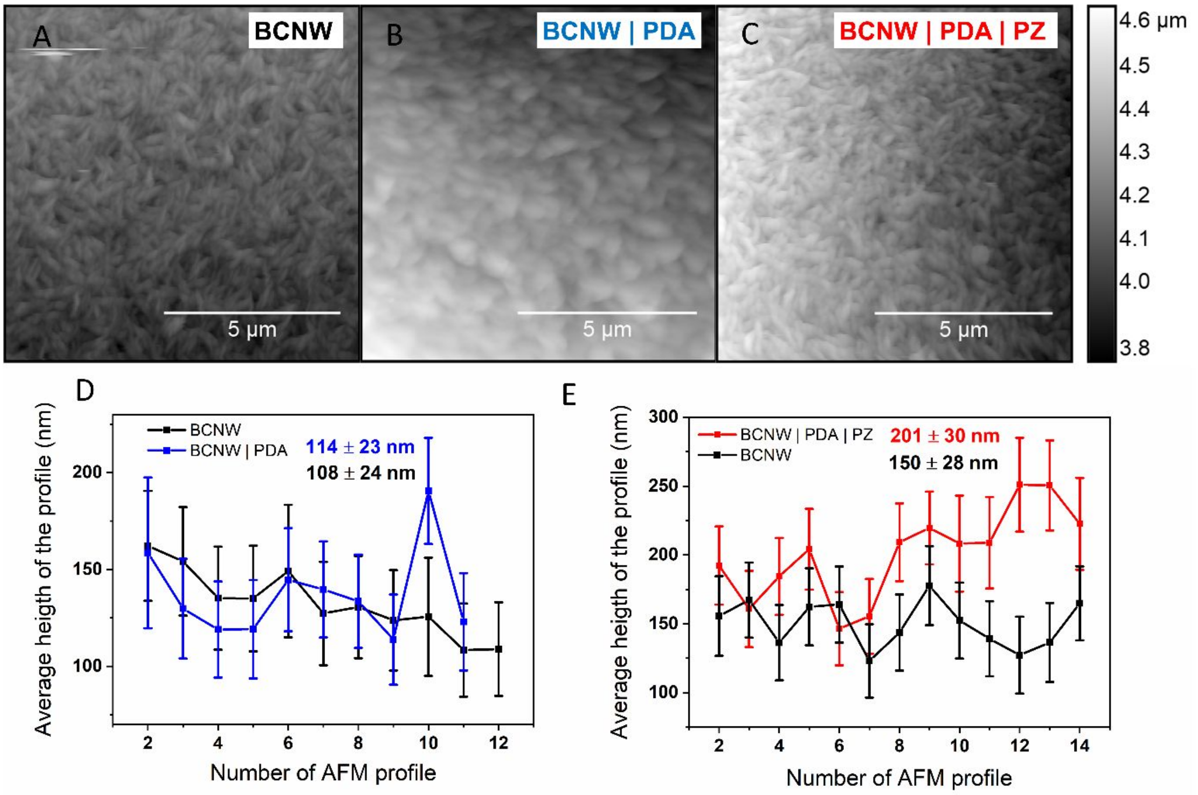

Figure S3. A-C AFM images of BCNW, BCNW | PDA and BCNW | PDA | PZ respectively; D-E histograms of the averaged AFM height obtained from several topographic scans for BCNW | PDA and BCNW | PDA | PZ respectively

Table S1. Nanomechanical properties calculated from indentation load-displacement curves in Figure 3B.

	BCNW	BCNW | PDA	BCNW | PDA | PZ
Reduced Young's modulus [GPa]	6.89	12.48	50.28
Hardness [GPa]	0.67	0.79	1.48
Elastic depth [nm]	178	106	35
Plastic depth [nm]	520	475	341
Elastic work [nJ]	0.74	0.45	0.18
Plastic work [nJ]	0.95	0.93	0.46

Table S2. Physiochemical quantities obtained via nonlinear fitting of EIS data and secondary quantities derived from those data (standard errors are given in brackets).

Element	BCNW	BCNW-PDA	BCNW | PDA | PZ

Quantities obtained via non-linear fitting			
Rs [Ω cm^2]	7.07 (0.3%)	13.2 (2.5%)	11.3 (0.2%)
Rct [Ω cm^2]	1.22 (3.6%)	81.40 (9.9%)	5.06 (1.1%)
Q_0[μF cm^{-2} s^{n-1}]	1457 (5.1%)	110 (3.2%)	667 (1.9%)
α [-]	0.67 (0.9%)	0.89 (0.6%)	0.70 (0.4%)
A_w [Ω cm^2 $s^{-1/2}$]	38.53 (1.2%)	524.2 (1.4%)	64.40 (0.6%)
χ^2	10e-5	2.3e-3	4e-5
Calculated quantities			
D [cm^2/s]	7.24e-6	0.29e-6	2.59 -6
k_0 [cm/s]	8.7e-2	0.13e-2	2.1e-2
C_{dl} (Brugg) [μF cm^{-2}]	61.10	47.80	48.73

Methods

Reagents

10X Tris Buffer was obtained from Santa Cruz Biotechnology and diluted 10 times prior to the measurements. Dopamine hydrochloride, sulfobetaine methacrylate (SBMA) and acetaminophen were purchased from Sigma-Aldrich. NaOH, KCl, HCl, glucose, ascorbic acid, glycine, $K_3Fe(CN)_6$ and $K_4Fe(CN)_6$, were obtained from Chempur. Hexaammineruthenium chloride (II) and (III) were purchased from Alfa Aesar. BSA crystals were acquired from SERVA. Deionized water used in the experiment was provided by a Hydrolab HLP-5p system.

SEM inspection

The morphology of the electrodes was investigated by means of a Quanta FEG 250 (FEI) Schottky field-emission scanning electron microscope (SEM) equipped with a secondary ET electron detector with a beam accelerating voltage of 10 kV.

XPS

The X-Ray Photoelectron Spectroscopy (XPS) studies were carried out on an Escalab 250Xi multispectroscope (ThermoFisher Scientific) operating with an AlKα X-ray source. The spot size was 650 μm and the pass energy through the hemispherical analyzer was 20 eV. Throughout the measurement, the samples were flooded with low-energy electrons and low-energy Ar+ ions to assure charge compensation, with a final peak calibration at adventitious carbon C 1s (284.8 eV). The obtained

spectra were analyzed and deconvoluted using the Avantage v5.9921 software (ThermoFisher Scientific).

FTIR characterization

The Fourier transform infrared spectra of the electrodes were registered using a Nicolet iS50 FTIR spectrometer equipped with a single-reflection diamond attenuated total reflectance (ATR) accessory ATR Special Quest. Spectra were recorded within the wavenumber range of 4,000–400 cm^{-1}. Analysis of the spectral data was performed using the OMNIC software.

Nanoindentation measurements

Five indentations separated by 100 μm were made for each electrode with a Berkovich indenter. Each measurement was performed at a maximum load of 5.0 mN with a 5 s dwell at maximum load. The loading and unloading rate was equal to 0.10 mN/s. To minimize the measurement errors, the tests were performed in a room with a temperature of 20 ± 0.5°C. Thermal drift correction was enabled with a 30 s data collection time. The correction was calculated using post-indentation drift calibration. Hardness and Young's modulus were calculated by the Oliver-Pharr method.

The multiple impulse experiment was performed with a pulse load of 5 mN and a stop load limit of 1.0 mN. One cycle consisted of a five-second load period and a two-second unloaded period. Ten pulses were delivered with a total experiment time of 70 seconds using the Berkovich indenter. The scratch test was performed with a load ranging from 0 to 100 mN, at a loading rate of 0.5 mN/s. During the experiment, data on acoustic emission and friction were collected. Each experiment was repeated three times. The scan length and scan velocities were 1000 μm and 3 μm/s, respectively.

Electrochemical Characterization

All voltammetric and impedance measurements were performed using a BioLogic SP-150 potentiostat-galvanostat in a standard three-electrode configuration at room temperature. Ag | AgCl | 3 M KCl was used as the reference electrode and a platinum mesh as the counter electrode, respectively. The geometric surface areas of the pristine and functionalized BCNW working electrodes were 0.5–1 mm^2.

The electrolyte for the cyclic voltammetry (CV), electrochemical impedance spectroscopy (EIS) and chronoamperometry (CA) measurements in the presence of ferrocyanides (Figure 4) was 5 mM $K_3Fe(CN)_6$ + 5 mM $K_4Fe(CN)_6$ + 1 M KCl. The frequency range for the EIS was 100 kHz – 100 mHz with 16 points per decade and 10 mV RMS potential. Before each EIS measurement, working electrodes were polarized for 3 minutes to +0.26 V, i.e., the formal potential of ferrocyanides. The EIS data were fitted to the electrical equivalent circuit using the Powell algorithm in the EIS Spectrum Analyzer software.[1] The electrolyte for CV measurements in the presence of hexaammineruthenium couple

consisted of 1 mM $K_3Ru(Cl)_6$ + 5 mM $K_4Ru(CN)_6$ + 1 M KCl. Analogous CV measurements in the presence of dopamine (Figure 5) consisted of 0.2 mM dopamine + 1 X Tris.

Difference pulsed voltammetry (DPV) detection of dopamine was performed with a 50 mV pulse height, 10 mV step height, 20 ms pulse width and 500 ms step time in the potential range from -0.2 V to +0.3 V (Figure 6). The electrolyte contained 0.1–100 µM of dopamine in 1x Tris. Limit of detection (LOD) was calculated according to the formula: $LOD = 3.3S_y/S$, where S_y is the standard deviation of the current response at the lower boundary of the linear range and S is the slope of this linear range.[2]

Different pHs for sensing were achieved by adding 1 M HCl or 1 M NaOH to the electrochemical cell, and controlled with an MP-103 handheld potentiometric pH meter. Bovine serum albumin (BSA) solution was prepared by dissolution of BSA crystals in 1x Tris solution so that the mass concentration was equal to 2%. The pH of this solution varied between 5 and 6. Consecutive amounts of dopamine were added in the range from 200 nM to 20 µM. After reaching 20 µM of interferants, i.e., glucose, ascorbic acid, glycine, and paracetamol were added subsequently at 60 µM each. After each addition, a DPV measurement was performed. Stability measurements (Figure 6 K) were performed by applying 55 consecutive DPV impulses and registering the current density peaks. Long-term measurements (Figure 6 L) were conducted in 2% BSA solution containing interferants for 7 days. After each 24 hour cycle, the DPV pulse was registered.

DFT calculations

The atomic structures of the PDA units were designed using a builder tool provided by Atomistic ToolKit Quantumwise (ATK, Synopsys, USA) as reported in [3]. Intermolecular interactions between the dopamine molecule and the zwitterions were investigated by performing geometry optimizations and calculating electron density maps on optimized structures. Density functional theory (DFT) on the generalized gradient approximation (GGA) level of theory with the Perdew–Burke–Ernzerhof (PBE) functional was applied as implemented in the package. The Linear Combination of Atomic Orbitals (LCAO) method[4] with high ATK basis set and PseudoDojo norm-conserving pseudopotentials were applied.[5] A pre-optimization using a Dreiding force field was used to facilitate several geometry optimizations.[6]

(1) Bondarenko A. S.; Ragoisha G. A. In Progress in Chemometrics Research; Pomerantsev A. L., Ed.; Nova Science Publishers: New York, 2005, Pp. 89–102 (the Program Is Available Online at Http://Www.Abc.Chemistry.Bsu.by/vi/Analyser/.

(2) Konieczka, P.; Namieśnik, J. *Quality Assurance and Quality Control in the Analytical Chemical Laboratory: A Practical Approach*, 2nd ed.; CRC Press, 2018. https://doi.org/10.1201/9781315295015.

(3) Atomistix Toolkit Version 2019.03, Synopsys QuantumWise A/S (Www.Quantumwise.Com), (n.d.).

(4) Soler, J. M.; Artacho, E.; Gale, J. D.; García, A.; Junquera, J.; Ordejón, P.; Sánchez-Portal, D. The SIESTA Method for *Ab Initio* Order- *N* Materials Simulation. *J. Phys.: Condens. Matter* **2002**, *14* (11), 2745–2779. https://doi.org/10.1088/0953-8984/14/11/302.

(5) van Setten, M. J.; Giantomassi, M.; Bousquet, E.; Verstraete, M. J.; Hamann, D. R.; Gonze, X.; Rignanese, G.-M. The PseudoDojo: Training and Grading a 85 Element Optimized Norm-Conserving Pseudopotential Table. *Computer Physics Communications* **2018**, *226*, 39–54. https://doi.org/10.1016/j.cpc.2018.01.012.

(6) Mayo, S. L.; Olafson, B. D.; Goddard, W. A. DREIDING: A Generic Force Field for Molecular Simulations. *J. Phys. Chem.* **1990**, *94* (26), 8897–8909. https://doi.org/10.1021/j100389a010.
